# CloneCNA: detecting subclonal somatic copy number alterations in heterogeneous tumor samples from whole-exome sequencing data

**DOI:** 10.1186/s12859-016-1174-7

**Published:** 2016-08-19

**Authors:** Zhenhua Yu, Ao Li, Minghui Wang

**Affiliations:** 1 School of Information Science and Technology, University of Science and Technology of China, Hefei, AH230027 China; 2 Centers for Biomedical Engineering, University of Science and Technology of China, Hefei, AH230027 China

**Keywords:** Copy number alteration, Intra-tumor heterogeneity, Whole-exome sequencing, Hidden Markov model

## Abstract

**Background:**

Copy number alteration is a main genetic structural variation that plays an important role in tumor initialization and progression. Accurate detection of copy number alterations is necessary for discovering cancer-causing genes. Whole-exome sequencing has become a widely used technology in the last decade for detecting various types of genomic aberrations in cancer genomes. However, there are several major issues encountered in these detection problems, including normal cell contamination, tumor aneuploidy, and intra-tumor heterogeneity. Especially, deciphering the intra-tumor heterogeneity is imperative for identifying clonal and subclonal copy number alterations.

**Results:**

We introduce CloneCNA, a novel bioinformatics tool for efficiently addressing these issues and automatically detecting clonal and subclonal somatic copy number alterations from heterogeneous tumor samples. CloneCNA fully explores the log ratio of read counts between paired tumor-normal samples and tumor B allele frequency of germline heterozygous SNP positions, further employs efficient statistical models to quantitatively represent copy number status of tumor sample containing multiple clones. We examine CloneCNA on simulated heterogeneous and real tumor samples, and the results demonstrate that CloneCNA has higher power to detect copy number alterations than existing methods.

**Conclusions:**

CloneCNA, a novel algorithm is developed to efficiently and accurately identify somatic copy number alterations from heterogeneous tumor samples. We demonstrate the statistical framework of CloneCNA represents a remarkable advance for tumor whole-exome sequencing data. We expect that CloneCNA will promote cancer-focused studies for investigating the role of clonal evolution and elucidating critical events benefiting tumor tumourigenesis and progression.

**Electronic supplementary material:**

The online version of this article (doi:10.1186/s12859-016-1174-7) contains supplementary material, which is available to authorized users.

## Background

Cancer is a dynamic disease featured by various genetic alterations that accumulate during the procedure of tumor development. The theory of clonal evolution [1] states that once a single precursor cell is initiated, the proceeding of neoplastic proliferation can to some extent be considered as a natural selection process – sequential selection by an evolutionary process. Over time, both similar and divergent genetic alterations beneficial for tumor persistence and growth are acquired by different tumor cells through clonal expansions. This results in the emergence of variant cell populations in tumors with each cell population containing a distinct complement of genetic alterations, which is known as the intra-tumor heterogeneity [1–3]. These genetic alterations consist of clonal aberrations, which derive from the precursor cell and exist in all tumor cells, and subclonal aberrations acquired during clonal expansions. Specifically, copy number alteration (CNA) has emerged as one of the main categories of genetic structural variations that plays an important role in tumor progression [4].

Deciphering the intra-tumor heterogeneity is imperative for identifying clonal and subclonal CNAs and further discovering cancer-causing genes. Associated studies [5–8] have benefited from continuous advances in experimental technologies [9–13] that are used for high-throughput profiling of cancer genomes. The recent next-generation sequencing (NGS) platforms, such as whole-genome sequencing (WGS) and whole-exome sequencing (WES), allows an unprecedented view of cancer genomes with nucleotide resolution. With the improvement in reliability and decrease in costs, WES has been considered as an effective alternative to WGS for CNA detection in tumors [14, 15]. However, such analysis is usually complicated by several critical issues encountered in interpretation of tumor WES data. First, the variable efficiency of exome capture results in non-uniform read depth between exome regions, and the read depth is simultaneously affected by the GC-content and length of individual exon [16]. These biases along with the discrete nature of the capture make WES unsuitable for whole-genome CNA detection methods. Second, tumor sample is usually contaminated by normal stroma and thus a mixture of tumor and non-tumor cells [17]. Here we use *tumor purity* to denote the proportion of cancerous cells in the tumor sample. Normal cell contamination will inevitably attenuate sequencing-derived copy number signals of aberrant exome regions. This sometimes makes it intractable to distinguish aberrant exome regions from normal regions, and eventually extract the aberration information from miscellaneous signals. Third, various numerical and structural chromosomal aberrations result in aneuploidy of tumor genomes, and the actual *tumor ploidy* is usually unknown [17–19]. Another critical issue comes from the fact that the tumor cell population may be heterogeneous, resulting from ongoing subclonal evolution [20], and the underlying number of distinct cell populations is unknown. Traditional CNA detection methods that make assumption of tumor homogeneity, i.e. mixture of normal and one tumor population, may omit CNAs that are present in only minor cell populations.

Critically, some of the aforementioned issues are strongly intertwined and cannot be solved separately, which may significantly complicate interpretation of WES data if they arise in the same tumor sample. For example, a gain of two copies of a chromosomal region in a tumor sample with 50 % normal cells could also be explained as a gain of one copy in a 100 % tumor sample. CNA detection methods from WES analyze target-normalized read counts or (log) ratio of read counts in exon, and detect deviations in copy number profiles along the exome to identify CNAs. Although several state-of-the-art computational methods [5, 6, 14, 15, 21–23] have greatly improved accuracy of the detected CNAs, their performance may still be limited by the aforementioned critical issues. For example, ExomeCNV [15] uses circular binary segmentation (CBS) method [24] to subdivide the exome and identify CNAs with correction for normal cell contamination, and EXCAVATOR [14] adopts a heterogeneous shifting level model (SLM) [25] to segment the exome and uses a classification method to identify CNAs, but tumor aneuploidy and intra-tumor heterogeneity are not explicitly modeled in these approaches. Control-FREEC [21] first infers the copy numbers of genomic segments, then uses Gaussian mixture model (GMM) to identify tumor genotypes with correction for both normal cell contamination and tumor aneuploidy. However, it does not take into account the issue of intra-tumor heterogeneity. THetA [5, 6] infers cancer subclones and detects subclonal CNAs in heterogeneous tumors using a delicate statistical model, but the number of underlying tumor clones is not automatically determined [26] and an integrated pipeline is not provided for segmentation analysis [22]. Taken together, there is great demand for developing sophisticated methods for CNA detection from tumor WES data by addressing all of the aforementioned challenges.

In this study, we introduce CloneCNA, an efficient bioinformatics tool for automatically detecting clonal and subclonal somatic CNAs using WES data of heterogeneous tumor samples. CloneCNA fully explores the log read counts ratio (LCR) between paired tumor-normal samples and tumor B allele frequency (BAF) of germline heterozygous SNP positions, then employs efficient statistical models to quantitatively represent copy numbers of tumor containing multiple clones. We adopt a factorial hidden Markov model (HMM) for jointly delineating aberration events and clonal clusters, and implement a model selection module based on Bayesian information criterion (BIC) to automatically determine the number of distinct clonal clusters. We compare CloneCNA with four state-of-the-art methods on simulated heterogeneous and real tumor samples, and the results demonstrate that CloneCNA has high power to detect both high and low cellularity CNA events.

## Methods

### The statistical model in CloneCNA

To depict tumors containing multiple subclones, we assume that the observed copy number profiles result from contributions of underlying three distinct cell populations: normal (non-cancerous) cells, cancerous cells with normal genotype, and cancerous cells harboring the aberration event. Thus, all cells can be further divided into two parts by a somatic aberration event at each locus: one with normal genotype and another containing the somatic aberration. We further assume multiple co-occurring events at different genomic loci share same cellularity, and can be designated to one of *K* clonal clusters. Here, a clone is defined as a cell population that is uniquely identified by a complement of aberration events, and the cellularity of an aberration is defined as the proportion of cells harboring the aberration. In addition, we use *β*_1:*K*_ to denote the cellularity of the *K* clonal clusters.

**Fig. 1:**
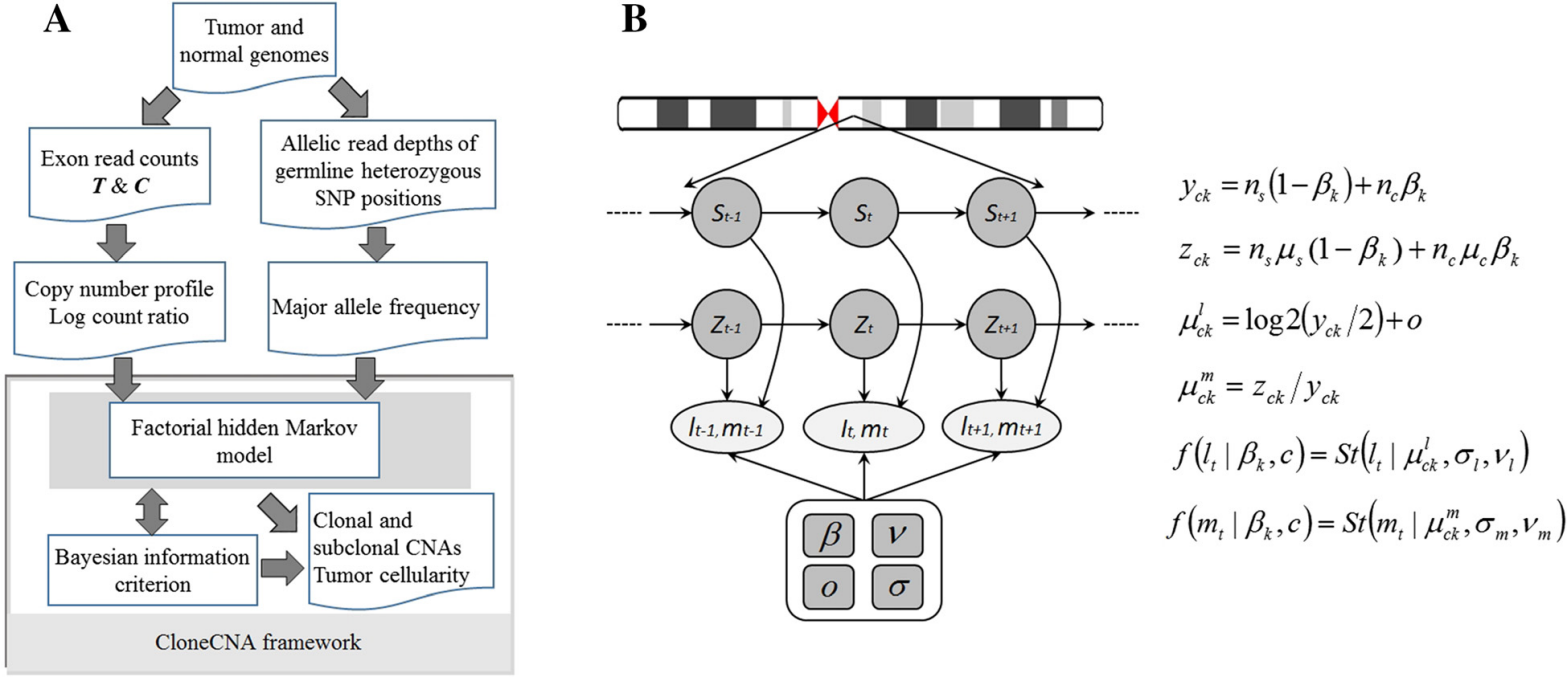
Overview of the CloneCNA probabilistic framework. **a** CloneCNA analysis workflow. Three inputs are required: 1) exon-level read counts of paired tumor-normal samples; 2) tumor allelic read depths of germline heterozygous SNP positions; 3) GC-content of all exon regions. The LCR and MAF data is modeled using a factorial HMM. The number of clonal clusters is determined using BIC. **b** HMM adopted in CloneCNA. Two Markov chains are adopted to delineate copy number aberrations and clonal clusters

The workflow is illustrated in Fig. 1a. The inputs to the model include exon-level read counts of paired tumor-normal samples, tumor allelic read depths of germline heterozygous SNP positions, and GC-content of all exon regions. All the inputs are obtained by using an in-house tool. Reads aligned to each exon are counted and sample-normalized for tumor and normal samples respectively, and the read counts ratio (CR) is calculated for each exon, which eliminate the inherent biases induced by difference in exon size and efficiency of exome capture. We assume that the paired tumor-normal samples are processed in the same way, i.e. samples are sequenced in same platform, and reads are processed under same configuration. Following our previous study [19], we adopt a non-parametric approach to normalize the CR for GC-content by using the following formula:

1 $$ {\tilde{r}}_i={r}_i\cdot m/{m}_X $$

where *r̃*_*i*_ is the normalized CR and *r*_*i*_ is the original CR of the *i*th exon, *m* denotes the median CR of all exons and *m*_*X*_ represents the median CR of the exons that have the same GC-content as the *i*th exon. The logarithm of the normalized CR (LCR) is then calculated to represent copy number measurements, and denoted as *l*_1:*N*_ for *N* exons. In addition, germline heterozygous SNP positions are extracted from normal genome by using SAMtools [27]. BAF of each SNP position is represented by the ratio between the B allelic and total read depths derived from tumor genome, and for each exon, we evaluate the median value of major allele frequency (MAF):

2 $$ {m}_i=\underset{j}{median}\left( \max \left({b}_{ij},1-{b}_{ij}\right)\right) $$

where *b*_*ij*_ denotes the BAF value of the *j*th SNP within the *i*th exon.

We use an integrated HMM to jointly analyze LCR and MAF signals (Fig. 1b), and determine the number of underlying clonal clusters based on BIC.

#### Hidden Markov model

We introduce a state list to depict copy number aberration states of tumor genomes (Table 1). Given a copy number aberration state *c*, the mean values of total and major allele copy number associated with the *k*th clonal cluster are defined as follows:

**Table 1:** Copy number aberration states defined in CloneCNA

Id	Total copy number	Major copy number	Tumor genotypes	Aberration type
1	0	0	N/A	HOMD
2	1	1	A, B	HEMD
3	2	1	AB	NHET
4	2	2	AA, BB	NLOH
5	3	2	AAB, ABB	AHET
6	3	3	AAA, BBB	ALOH
7	4	2	AABB	AHET
8	4	3	AAAB, ABBB	AHET
9	4	4	AAAA, BBBB	ALOH
10	5	3	AAABB, AABBB	AHET
11	5	4	AAAAB, ABBBB	AHET
12	5	5	AAAAA, BBBBB	ALOH

Aberration types include homozygous deletion (HOMD), hemizygous deletion (HEMD), copy neutral heterozygosity (NHET), copy neutral LOH (NLOH), amplified heterozygosity (AHET) and amplified LOH (ALOH)

3 $$ {y}_{ck}={n}_s\left(1-{\beta}_k\right)+{n}_c{\beta}_k $$

4 $$ {z}_{ck}={n}_s{\mu}_s\left(1-{\beta}_k\right)+{n}_c{\mu}_c{\beta}_k $$

where *n*_*s*_ is the copy number of normal genomes, *n*_*c*_ is the tumor copy number associated with state *c*, *μ*_*s*_ represents the expected MAF of normal genomes, and *μ*_*c*_ denotes the expected MAF of tumor genotypes in state *c*. The mean values of LCR and MAF signals are then formulated with:

5 $$ {\mu}_{ck}^l= \log 2\left({y}_{ck}/2\right)+o $$

6 $$ {\mu}_{ck}^m={z}_{ck}/{y}_{ck} $$

The parameter *o* is introduced to account for the baseline shift of LCR signals and varies with respect to the change of tumor ploidy. The parameters *μ*^*l*^_*ck*_ and *μ*^*m*^_*ck*_ are thus the functions of *β*_1:*K*_ and therefore reflect the joint-effect on LCR and MAF signals from three types of cell populations, respectively. We assume *l*_1:*N*_ are Student’s t-distributed with the conditional probability density function defined as follows:

7 $$ f\left({l}_i\Big|c,k,{\sigma}_l,{\nu}_l,o\right)=\frac{\varGamma \left(\left({\nu}_l+1\right)/2\right)}{\varGamma \left({\nu}_l/2\right)\sqrt{\pi {\nu}_l}{\sigma}_l}{\left(1+\frac{1}{\nu_l}{\left(\frac{l_i-{\mu}_{ck}^l}{\sigma_l}\right)}^2\right)}^{-\frac{\nu_l+1}{2}} $$

where *ν*_*l*_ is the number of degrees of freedom, *σ*_*l*_ is the scale parameter, and Γ represents the gamma function. Similarly, we also assume *m*_1:*N*_ are Student’s t-distributed:

8 $$ f\left({m}_i\Big|c,k,{\sigma}_m,{\nu}_m\right)=\frac{\varGamma \left(\left({\nu}_m+1\right)/2\right)}{\varGamma \left({\nu}_m/2\right)\sqrt{\pi {\nu}_m}{\sigma}_m}{\left(1+\frac{1}{\nu_m}{\left(\frac{m_i-{\mu}_{ck}^m}{\sigma_m}\right)}^2\right)}^{-\frac{\nu_m+1}{2}} $$

The conditional probability densities of LCR and MAF signals depend on two latent variables, namely copy number aberration state *c* and clonal cluster *k*, therefore we implement CloneCNA as a HMM with *C* × *K* hidden states (Fig. 1b). Here, *C* is the number of copy number aberration states defined in Table 1 and *K* is the number of clonal clusters. The HMM is thus equivalent to a factorial HMM with 2 underlying Markov chains with one chain depicting aberrations and another delineating clonal clusters (Fig. 1b). For a given value of *K*, expectation maximization (EM) algorithm [28] is employed to estimate the model parameters *θ* = (*π*, *A*, *β*, *o*, *σ*_*l*_, *ν*_*l*_, *σ*_*m*_, *ν*_*m*_), where *π* represents initial state probability distribution, and *A* denotes state transition matrix. In the expectation step of the EM algorithm, the expectations of the partial log-likelihood functions of LCR and MAF are formulated as:

9 $$ E\left(L{L}_l\right)={\displaystyle \sum_{i=1}^N{\displaystyle \sum_{c=1}^C{\displaystyle \sum_{k=1}^K{\gamma}_{ick} \log \left(f\left({l}_i\Big|c,k,{\sigma}_l,{\nu}_l,o\right)\right)}}} $$

10 $$ E\left(L{L}_m\right)={\displaystyle \sum_{i=1}^N{\displaystyle \sum_{c=1}^C{\displaystyle \sum_{k=1}^K{\gamma}_{ick} \log \left(f\left({m}_i\Big|c,k,{\sigma}_m,{\nu}_m\right)\right)}}} $$

We use forward-backward algorithm [29] to calculate the posterior probability *γ*_*ick*_ that the *i*th exon is in state *c* and clonal cluster *k*. In the maximization step, Newton–Raphson method [30] is used to iteratively update model parameters.

The parameter updating procedure is stopped when the EM algorithm converges. The copy number aberration state and clonal cluster of each exon are determined by the hidden state associated with the maximum posterior probability. Segmentation of the exons is then performed to output copy numbers and cellularity of all segments. Moreover, for each segment, a reliability score is calculated based on observed probability densities to evaluate the reliability of CloneCNA results (Additional file 1). We also adopt a grid search of *θ* to find the optimal solution that give the maximum log-likelihood value of LCR and MAF data.

#### Model selection

The hidden state space of the HMM in CloneCNA is expanded as a function of the number of clonal clusters *K*, which is determined under the BIC in the CloneCNA framework. We aim to find an optimal value of *K* that leads to the model associated with the lowest value of BIC. Starting with the initial assumption of tumor homogeneity (*K* = 1), CloneCNA iteratively increases the number of clonal clusters by one until the BIC of the model no longer decreases or the allowed maximum number of clonal clusters (10) is reached. We provide a detailed description of the model selection procedure in the Additional file 1.

### Real WES data of tumor samples

WES data from 9 paired primary triple negative breast cancer (TNBC) samples [31] is used in this study. Reads are sequenced to approximately 30× coverage on Illumina Genome Analyzer IIx platform and mapped to the reference genome NCBI36/hg18 using BWA [32]. We download the data from European Genome-Phenome Archive (EGA) under accession number EGAS00001000132.

### Simulated heterogeneous tumor samples

Four test tumor genomes (denoted as *t*1, *t*2, *t*3 and *t*4) are generated to simulate multiple tumor clonal populations by mixing different combinations of genomes at predefined proportions. We use the WES data of a real normal sample to generate sequencing data of the test genomes (Additional file 2: Figure S1). Each test genome is constructed by following four steps: 1) divide the reference genome into a series of segments, each segment is then assigned with a specific genomic aberration defined by total copy number and major allele copy number, 2) randomly sample reads from the normal genome according to the copy number of each segment of the test genome, 3) further process the sampled reads to match the BAF of SNPs within each segment, and 4) merge and process the modified reads using SAMtools [27] to generate BAM. In addition, a normal genome (denoted as *n*) is also constructed by following the same procedure to simulate normal cell contamination in tumor samples. The detailed aberration information of each genome is provided in (Additional file 3: Table S1). For each combination of samples, reads are sampled at known proportion from BAM files to generate the mixture. By this way, we totally generate 20 heterogeneous tumor samples, of which each mixture is derived from a specific combination of the normal and four tumor genomes (Additional file 4: Table S2). With these simulated samples, we perform a comprehensive evaluation of CloneCNA in terms of detecting clonal and subclonal somatic CNAs.

### Competitive methods

In evaluating performance of CloneCNA, four state-of-the-art methods for CNA detection using WES data, i.e. ExomeCNV [15], Control-FREEC [21], EXCAVATOR [14], and THetA [6], are adopted to make comparison between CNA calling methods. The detailed description of performance evaluation strategy and investigated methods is provided in Additional file 1.

## Results

### GC-content correction of LCR data

We first investigate the effect of GC-content on the LCR signals, and assess the capability of GC-content correction procedure. The LCR values and GC-content of exons with copy number ranging from 1 to 3 are analyzed respectively, and the results are shown in (Additional file 2: Figure S2). Our analysis indicate that the LCR signals demonstrate a significant correlation with GC-content (correlation coefficient = 0.32, 0.29 and 0.34 for 1–3 copies respectively), and require a normalization procedure before being used to identify aberrant exome regions. Based on our previous study [19], we then implement a normalization procedure for the removal of GC-content bias (see Methods for more details). After normalization (Additional file 2: Figure S2B), the GC-content effect is significantly eliminated (correlation coefficient = 0.09, 0.03 and 0.06 for 1–3 copies respectively).

### Results on simulated data

We first investigate the LCR and BAF distributions associated with different types of aberrations to analyze the influence of normal cell contamination for WES data and the results are shown in Additional file 2: Figure S3. When tumor purity decreases, it is observed that for each aberration both the LCR and BAF signals gradually approach close to the expected values of normal genotype (0.5 for BAF and 0 for LCR). These results imply that normal cell contamination will inevitably diminish aberrant signals and needs to be corrected for accurately inferring genomic aberrations.

**Fig. 2:**
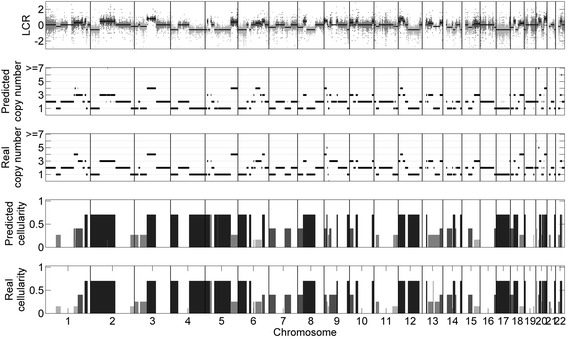
CNA detection and cellularity estimation results of CloneCNA on a simulated sample. The CNAs predicted by CloneCNA are significantly consistent with the underlying ground truth. At the same time, CloneCNA correctly estimates the number of clonal clusters and their cellularity with 0.16, 0.27, 0.4 and 0.7

We apply CloneCNA to 20 simulated samples to examine its ability of predicting cellularity of distinct clonal clusters and detecting CNAs. An example of the results on a simulated sample is shown in Fig. 2. Four tumor genomes (*t*1, *t*2, *t*3 and *t*4) and the simulated normal genome (*n*) are mixed to generate the simulated sample with corresponding proportion of 0.3, 0.15, 0.1, 0.15 and 0.3 respectively. This results in the emergence of four clonal clusters with different cellularity of 0.15, 0.25, 0.4 and 0.7 (see details in Additional file 1). The results show that CloneCNA correctly estimates the number of clonal clusters and infers their cellularity (0.16, 0.27, 0.4 and 0.7), meanwhile CNAs represented in each clonal cluster are well identified. CloneCNA exhibits similar performance on other simulated tumor samples (data is not shown).

To assess the abilities of different methods in detecting clonal CNAs, we first apply the five methods to homogeneous tumor samples. The tumor purity of these samples ranges from 0.1 to 0.9. The CNA states of all exons are used as the golden standard to evaluate different computational methods, and two metrics, sensitivity and specificity, are measured for each sample and method (see details in Additional file 1). The results of five methods, ExomeCNV, Control-FREEC, EXCAVATOR, THetA and CloneCNA, are shown in Additional file 2: Figure S4. ExomeCNV, Control-FREEC and EXCAVATOR present good specificity across all samples, and meanwhile ExomeCNV and Control-FREEC shows a generally higher sensitivity than other existing methods. In addition, all the four methods show excellent performance for detecting CNAs when tumor purity is greater than 0.7. By Comparison, CloneCNA demonstrates strong robustness to tumor purity and keeps high sensitivity (>0.94) with tumor purity greater than 0.1. It also maintains comparable high specificity (>0.96).

**Fig. 3:**
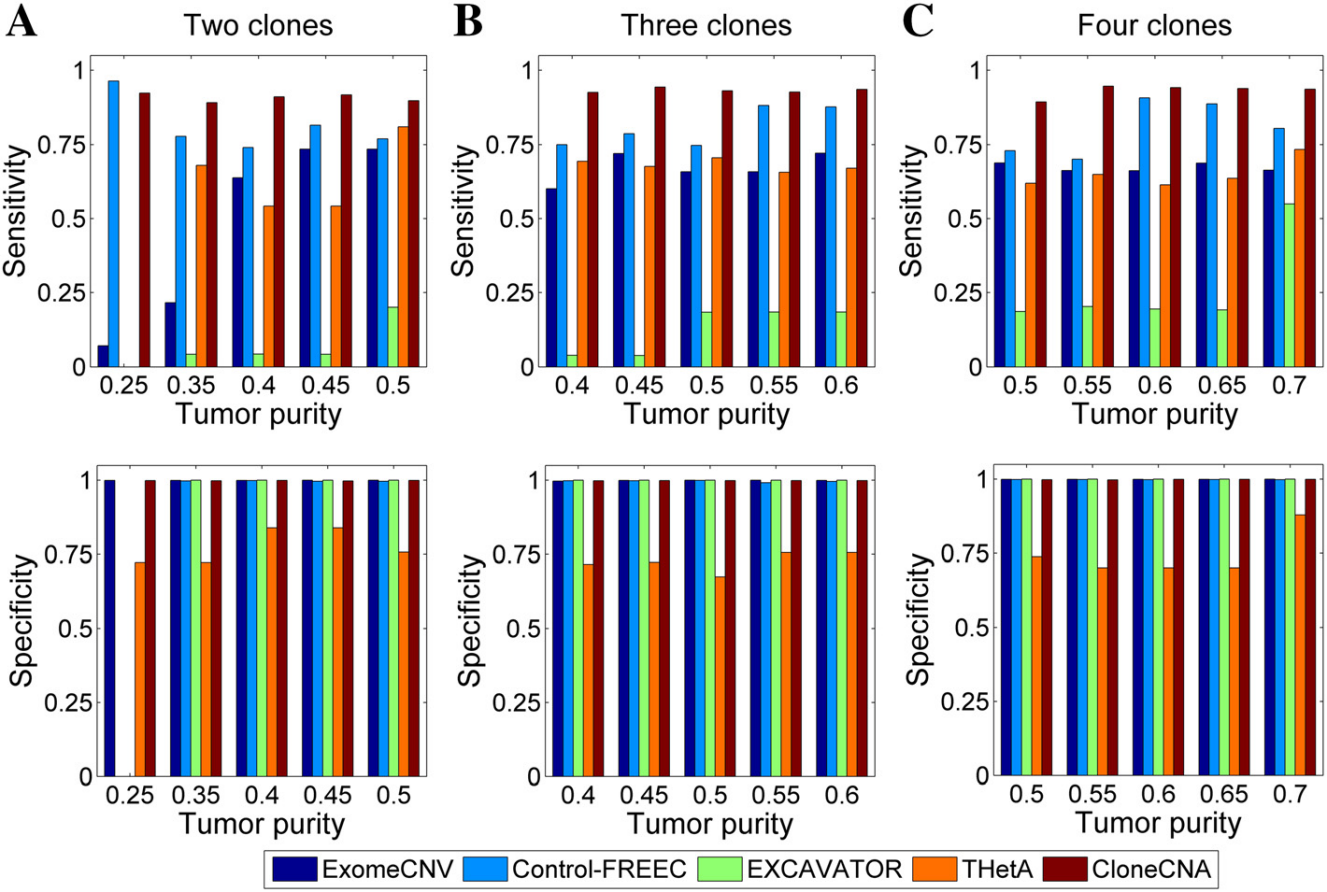
CNA detection performance of ExomeCNV, Control-FREEC, EXCAVATOR, THetA and CloneCNA on simulated heterogeneous samples. Sensitivity and specificity are calculated for each sample based on the results of each investigated method. **a** Results for samples containing two clonal populations. For the sample with tumor purity of 0.25, EXCAVATOR reports a running error, therefore the performance of EXCAVATOR and THetA on this sample is not evaluated. **b** Results for samples containing three clonal populations. **c** Results for samples containing four clonal populations

Next, we proceed to evaluate the performance of each method for detecting clonal and subclonal CNA events in samples containing multiple tumor subclones. For this purpose, we simulate 15 samples containing two, three or four clonal populations (Additional file 4: Table S2). The sensitivity and specificity of each method are shown in Fig. 3. Similar to the case observed for homogeneous tumor samples, ExomeCNV, Control-FREEC and EXCAVATOR consistently achieve high specificity (>0.99) nearly in all samples. On the other hand, ExomeCNV and THetA perform similarly in identifying CNAs with a median sensitivity of 0.66 and 0.68 respectively, Control-FREEC achieves a higher median sensitivity of 0.8. In comparison, CloneCNA keeps consistent high sensitivity (>0.89) for detecting either clonal or subclonal CNAs, and shows remarkable advantage when compared with other methods. It also gets comparable high specificity (>0.99) across all samples. These results indicate that CloneCNA can provide accurate identifications for clonal and subclonal CNAs.

We further assess the performance of copy number prediction of investigated methods, and the results are shown in Additional file 2: Figure S5. The copy number states of all exons are used to make comparison. Among existing methods, ExomeCNV and Control-FREEC generally perform better than other methods, and achieve median accuracies of 0.78 and 0.83, respectively, while EXCAVATOR and THetA have median accuracies of 0.57 and 0.55. By comparison, CloneCNA demonstrates high accuracy for predicting copy numbers with median accuracy of 0.94.

To evaluate the accuracy of cellularity predictions of CloneCNA, we make a comparison between the estimated cellularity and the underlying ground truth cellularity for each simulated sample. For each simulated sample, the underlying cellularity is computed by using contribution from each tumor genome making up the mixture (see details in Additional file 1). CloneCNA accurately estimates the cellularity with highly significant positive correlation (correlation coefficient > 0.99, *p* < 1×10^−5^, and mean absolute error < 0.03) with the ground truth cellularity across all mixed samples (Additional file 2: Figure S6), indicating CloneCNA can precisely reproduce clonal components. The number and cellularity of underlying clonal clusters for all mixtures are provided in (Additional file 5: Table S3). Moreover, we evaluate the accuracy of tumor purity estimations of CloneCNA, and the results show statistically significant correlation for the all simulated mixtures (correlation coefficient = 0.99, *p* = 2.99×10^−26^, mean absolute error = 0.008) relative to the underlying tumor purity (Additional file 2: Figure S7).

### Results on real data

Having demonstrated the validity of CloneCNA on simulated data, we proceed to examine the performance of CloneCNA on 9 TNBC paired tumor-normal samples. The TNBC samples are also assayed by Affymetrix SNP6.0 genotyping arrays. We use results from ASCAT [33] software based on the SNP array data as a baseline for comparison between different methods. Details on investigated tools and performance evaluation strategy are described in Additional file 1.

We first evaluate the tumor purities of the TNBC samples using four algorithms – Control-FREEC, THetA, CloneCNA and ASCAT, and the results are summarized in Table 2. The tumor purities estimated by ASCAT are used as the ground truth to make a comparison between other algorithms. We find CloneCNA is more accurate with mean absolute error (MAE) of 0.07 when compared with Control-FREEC (MAE = 0.24) and THetA (MAE = 0.09). The good concordance with the ground truth underscores the ability of CloneCNA in reliably inferring the tumor purity from WES of complicated tumor samples.

**Table 2:** Tumor purity estimated by Control-FREEC, THetA, CloneCNA and ASCAT for TNBC samples

Sample	Control-FREEC	THetA	CloneCNA	ASCAT
SA018	0.78	0.67	0.61	0.63
SA029	0.84	0.6	0.44	0.48
SA030	0.87	0.51	0.44	0.47
SA031	0.26	0.51	0.36	0.41
SA051	0.3	0.5	0.38	0.44
SA052	0.84	0.62	0.84	0.51
SA065	0.96	0.49	0.66	0.67
SA069	0.55	0.5	0.41	0.42
SA071	0.94	0.63	0.66	0.72
MAE	0.24	0.09	0.07	N/A

**Fig. 4:**
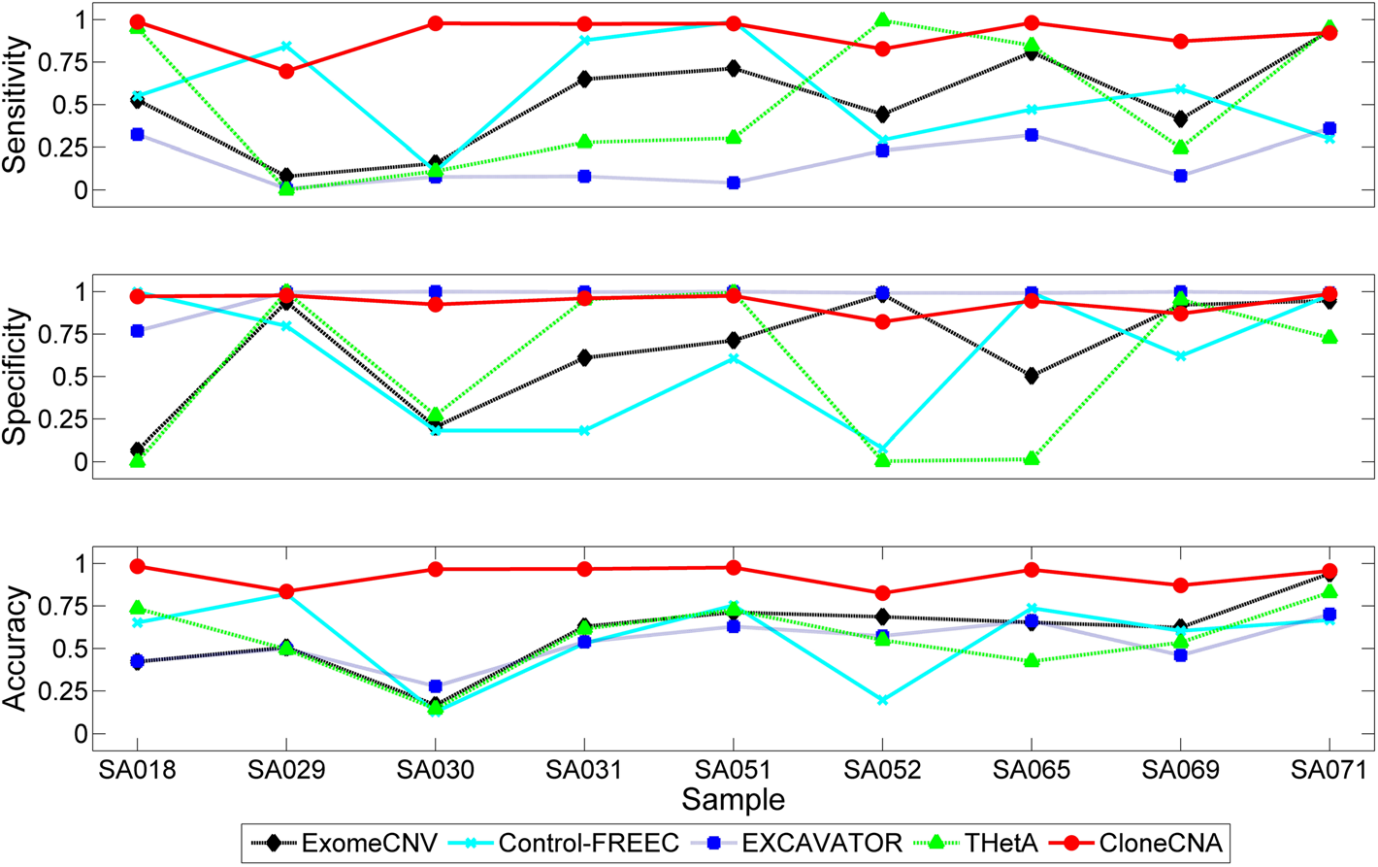
CNA detection performance of ExomeCNV, Control-FREEC, EXCAVATOR, THetA and CloneCNA on TNBC samples. CNAs detected by ASCAT from Affymetrix SNP6.0 arrays are used as ground truth for comparing different methods. Sensitivity, specificity and accuracy of all methods on each sample are measured

Next, we evaluate the CNA detection performance of ExomeCNV, Control-FREEC, EXCAVATOR, THetA and CloneCNA on the TNBC samples (Fig. 4). For all samples, CloneCNA compares favorably to other methods and presents higher sensitivity in most of the samples, with specificity comparable to those of the other methods. The median values of accuracy of ExomeCNV, Control-FREEC, EXCAVATOR and THetA are 0.63, 0.65, 0.54 and 0.55 respectively. In comparison, CloneCNA achieves superior accuracy with median value of 0.96.

**Fig. 5:**
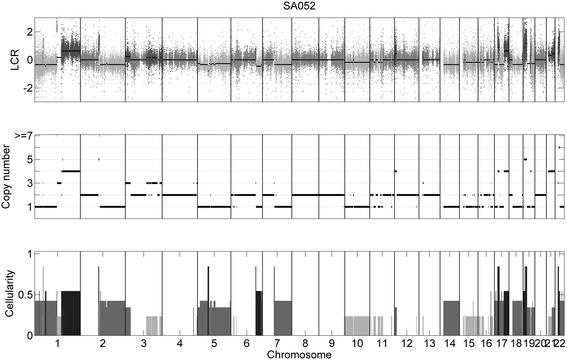
CNA detection and cellularity estimation results of CloneCNA on real sample SA052. CloneCNA identifies five clonal clusters with corresponding cellularity of 0.23, 0.34, 0.42, 0.54 and 0.84 respectively

Then, we proceed to investigate whether there exists multiple tumor subclones in the TNBC samples. From the results of ASCAT on sample SA052, we find a distinct mismatching between the mean of observed BAF signals and the expected BAF value for regions of homozygous and hemizygous deletions on chromosomes 5 and 10 (Additional file 2: Figure S8A), which indicates that there may be tumor subpopulations in the sample. Interestingly, in the CloneCNA analysis of the sample SA052, we find there are multiple clonal clusters. The log-likelihoods and BIC difference of the models associated with different number of clonal clusters are shown in Additional file 2: Figure S9. When the number of clonal clusters increases to 6, the BIC difference changes to positive, therefore CloneCNA finally infers *K* = 5 as the optimal number of clonal clusters. The cellularity of clonal clusters are 0.23, 0.34, 0.42, 0.54 and 0.84 respectively (Fig. 5). CloneCNA identifies board hemizygous deletions on chromosome 1p, 2q, 5, 7q, 10, 14 and 18q. These hemizygous deletions are represented in subclonal clusters. The difference of the BAF and LCR signals in hemizygous deletion regions represented in clonal and subclonal clusters is shown in Additional file 2: Figure S10. Moreover, ASCAT infers a homozygous deletion on chromosome 5, while CloneCNA predicts it as clonal hemizygous deletion, and the hemizygous deletions identified by ASCAT are almost represented in subclonal clusters from the results of CloneCNA (Additional file 2: Figure S8B).

Finally, we extract the mutations of TNBC samples that are validated in a previous study [31], and make mappings between the mutations and copy number detections of the CNA-calling methods (Additional file 6: Table S4). We separately count the number of mutations predicted to be in copy deleted, neutral and amplified regions by multiple methods, and the results are shown in Additional file 2: Figure S11. Our analysis show that for CNA regions simultaneously inferred by all methods, there are 35 copy amplified mutations, of which 4 mutations derive from sample SA052 (Additional file 7: Table S5). For two mutations, CloneCNA predicts similar clonality of 0.64 and 0.50 respectively when compared with the clonality (0.68 and 0.52) inferred from mutation level.

## Discussion

CloneCNA is a novel algorithm for detecting clonal and subclonal somatic CNAs from whole-exome sequencing data of heterogeneous tumor samples. It fully explores the log ratio of depth-of-coverage between paired tumor-normal samples and tumor allelic read depths of germline heterozygous SNP positions. A HMM is integrated in the framework of CloneCNA to depict copy number aberrations of tumors containing multiple clonal populations. Moreover, the underlying number of clonal clusters is determined under the BIC in the CloneCNA framework. Upon these specific features, CloneCNA presents advantages in several aspects. First, the appropriate deconvolution of miscellaneous signals of sequencing reads enables improved performance for detecting CNAs. Second, CloneCNA is much more sensitive to low cellularity clonal and subclonal CNA events when compared to existing methods, demonstrated by intra-tumor heterogeneity simulation experiment. Third, the algorithm provides accurate estimation of tumor purity. In summary, by benchmarking the performance of our algorithm on both simulated and real whole-exome sequencing data, we demonstrate that proper representation of the tumor WES data by considering intra-tumor heterogeneity can lead to more sensitive CNA detection power, and CloneCNA outperforms existing methods in detecting either high or low cellularity clonal and subclonal CNAs.

Despite the advantages mentioned above, CloneCNA has several limitations in analysis of tumor WES data due to its adopted modelling assumptions. First, CloneCNA simply assumes that, at each aberrant locus, there exists only one tumor genotype. This assumption will not hold if more than one aberrant genotypes exist at the same locus and represented in distinct tumor populations. However, it is much challenging to distinguish among multiple tumor clones that have variable aberrated genotypes. The statistical models for representation of LCR and MAF signals in CloneCNA need to be improved to account for multiple tumor genotypes, and the analysis of data may be confounded by multiple similar solutions. Second, CloneCNA is unable to identify the coexistence of distinct clones that are present at the same or similar cellularity within the sample. This is a common problem for most mixture separation methods. Third, one of the potential improvement of CloneCNA is to integrate somatic SNVs, we plan to further extend CloneCNA in this direction to provide more comprehensive “fingerprint” of a clonal expansion.

## Conclusions

Efficiently addressing critical issues such as normal cell contamination, tumor aneuploidy, and intra-tumor heterogeneity, is necessary for accurately identify somatic CNAs from heterogeneous tumor samples. In this study, we demonstrate that CloneCNA statistical framework represents a remarkable advance in detecting somatic CNAs for tumor whole-exome sequencing data. We expect that CloneCNA will promote cancer-focused studies for investigating the role of clonal evolution and elucidating critical events benefiting tumor tumourigenesis and progression.

## Additional files

Additional file 1: This file provides detailed description of CloneCNA statistical framework, performance evaluation strategy, simulation experiment, and details of the investigated methods. (PDF 605 kb) Additional file 2: Presentations of Supplementary Figures. (PDF 1712 kb) Additional file 3: Detailed aberration information of each simulated genome. (XLS 36 kb) Additional file 4: Simulated mixtures with combinations of four genomes. (XLS 26 kb) Additional file 5: The number and cellularity of underlying clonal clusters for all simulated mixtures. (XLS 28 kb) Additional file 6: Mappings between mutations and copy number detections of the CNA-calling methods. (XLS 65 kb) Additional file 7: Four amplified mutations in sample SA052. (XLS 23 kb)

## Abbreviations

CNA: Copy number alteration
SNP: Single nucleotide polymorphism
NGS: Next-generation sequencing
WGS: Whole-genome sequencing
WES: Whole-exome sequencing
HMM: Hidden Markov model
BIC: Bayesian information criterion
LCR: Log read counts ratio
BAF: B allele frequency
TNBC: Triple negative breast cancer
EGA: European genome-phenome archive
EM: Expectation maximization
