# Supplementary material for: CloneCNA: detecting subclonal somatic copy number alterations in heterogeneous tumor samples from whole-exome sequencing data
Source: BMC Bioinformatics. 2016 Aug 19;17:310. doi: 10.1186/s12859-016-1174-7 (PMC4990858; doi:10.1186/s12859-016-1174-7)
Supplement: Additional file 1: — This file provides detailed description of CloneCNA statistical framework, performance evaluation strategy, simulation experiment, and details of the investigated methods. (PDF 605 kb) [file 12859_2016_1174_MOESM1_ESM.pdf]

CloneCNA: detecting subclonal somatic copy number alterations in heterogeneous tumor samples from whole-exome sequencing data

Supplementary Material

Zhenhua Yu[1], Ao Li[1,2,*] and Minghui Wang[1,2]

[1]School of Information Science and Technology, University of Science and Technology of China, Hefei, AH230027, China.

[2]Centers for Biomedical Engineering, University of Science and Technology of China, Hefei, AH230027, China.

*To whom correspondence should be addressed. Email: aoli@ustc.edu.cn

Contents

1. Supplementary Methods 2
- 1.1. CloneCNA pipeline 2
- 1.2. CloneCNA statistical framework 2
- 1.2.1. Presentation of tumor heterogeneity in WES data 2
- 1.2.2. Hidden Markov Model 2
- 1.2.3. Bayesian Information Criterion for model selection 4
- 1.3. Reliability score for CNA detection 4
- 1.4. Performance evaluation 4
- 1.5. Simulation of intra-tumor heterogeneity 5
- 1.6. Details of the investigated methods 5
- 1.6.1. ExomeCNV 5
- 1.6.2. Control-FREEC 5
- 1.6.3. EXCAVATOR 5
- 1.6.4. THetA 5
- 1.6.5. CloneCNA 6

1. Supplementary Methods

1.1. CloneCNA pipeline

The inputs to CloneCNA include exon-level read counts of paired tumor-normal genomes, tumor allelic read depths of germline heterozygous SNP positions, and GC-content of all exon regions. Read counts of each exon is obtained by counting the reads with starting position within the exon region and further sample-normalized for the tumor and normal samples respectively, and the read counts ratio is calculated for each exon. The read counts ratio is further processed to correct GC-content bias as described in the main text. The logarithm of the corrected read counts ratio (LCR) is then calculated to represent copy number measurements of each exon, here we use $l_{1:N}$ to denote the LCR values of all exons. In addition, germline heterozygous SNP positions are fetched from the normal genome by using SAMAtools [1]. For B allele frequency, we first obtain total read depth by counting the reads mapping to each SNP, and non-reference (B allele) read depth is measured as the number of reads with non-reference base at the SNP, BAF of each SNP is thus represented by the ratio between the B allelic and total read depths. For each exon, the median value of major allele frequency (MAF) is calculated and we use $m_{1:N}$ to denote the MAF values of all exons. CloneCNA jointly analyzes $l_{1:N}$ and $m_{1:N}$ using an integrated hidden Markov model (HMM), and perform a grid search of the model parameters to find the optimal solution that give the maximum log-likelihood value of LCR and MAF data. To determine the underlying number of clonal clusters, we implement a model selection module in the framework based on Bayesian information criterion (BIC), and the procedure is represented as following: CloneCNA starts with the assumption of one clonal cluster and then iteratively increases clonal cluster count by one until the BIC value of the model no longer decreases. Segmentation of the exons is then performed to give the final results of copy numbers and cellularity of each segment.

1.2. CloneCNA statistical framework

1.2.1. Presentation of tumor heterogeneity in WES data

To depict tumor heterogeneity in WES data, we assume that the observed copy number profiles at a locus results from contributions of three distinct cell populations: normal (non-cancerous) cells, tumor cells with normal genotype, and tumor cells harboring the aberration event. We further assume that multiple co-occurring events at different loci will be represented in same clone, and all aberration events can be designated into a finite number of *K* clonal clusters with corresponding cellularity $\beta_{1:K}$. The copy number aberration states considered in this study are presented in Table 1.

Copy number is represented by the LCR values $l_{1:N}$, which are assumed to be Student's t-distributed with mean

$$\mu_{ck}^{l} = \log 2(y_{ck} / 2) + o \tag{1}$$

The average copy number y_{ck} associated with aberration state *c* and the *k*th clonal cluster is defined as:

$$y_{ck} = n_s(1-\beta_k) + n_c\beta_k \tag{2}$$

where n_s is the normal copy number and fixed to 2, n_c is the tumor copy number in aberration state *c*, and *o* is introduced to account for the baseline shift of LCR signals and varies with respect to the change of tumor ploidy.

Allelic imbalance is represented by the MAF values $m_{1:N}$, which are also assumed to be Student's t-distributed with mean

$$\mu_{ck}^{m} = z_{ck} / y_{ck} \tag{3}$$

where z_{ck} is the major allele copy number and formulated as:

$$z_{ck} = n_s\mu_s(1-\beta_k) + n_c\mu_c\beta_k \tag{4}$$

where μ_s is the expected MAF of normal genomes, and μ_c is the expected MAF of tumor genotypes in state *c*.

Note that for formula (2) and (4), our statistical model assumes that, at each aberrant locus, there exists only one common copy number aberration.

1.2.2. Hidden Markov Model

With combination of copy number aberrations and clonal clusters, we implement CloneCNA as a HMM with $C \times K$ hidden states, here *C* is the number of

copy number states defined in Table 1 and K is the number of clonal clusters. The HMM is thus equivalent to a factorial HMM with 2 underlying Markov chains with one chain depicting aberration event and another delineating clonal cluster (Figure 1B). The hidden state space is expanded as a function of the number of clonal clusters. Therefore, the model structure directly depends on the number of clonal clusters, which is determined by using BIC implemented in the framework.

The emission models of the HMM can be found in the main text. We further incorporate the effect of signal fluctuation in the emission models of CloneCNA, and in this case uniform distributions are employed to approximate the statistical distribution of LCR and MAF:

$$f\left(l_i \mid c=0,k\right)=\begin{cases}\dfrac{1}{b-a} & a\le l_i\le b\\ 0 & others\end{cases} \tag{5}$$

$$f\left(m_i \mid c=0,k\right)=\begin{cases}\dfrac{1}{f-e} & e\le m_i\le f\\ 0 & others\end{cases} \tag{6}$$

We employ the expectation maximization (EM) algorithm to estimate the model parameters

$$\theta=\left(\pi,A,\beta,o,\sigma_l,\nu_l,\sigma_m,\nu_m\right) \tag{7}$$

given the number of clonal clusters K. Where π is the initial state distribution, A is the transition probability matrix, β is the cellularity, o is the LCR baseline shift, σ_l and σ_m are scale parameters, ν_l and ν_m are the degrees of freedom of Student's t-distributions.

In the expectation step of the EM algorithm, the expectation of the partial log-likelihood functions of LCR and MAF are formulated as:

$$E(LL_l)=\sum_{i=1}^{N}\sum_{c=1}^{C}\sum_{k=1}^{K}\gamma_{ick}\log(f(l_i\mid c,k,\sigma_l,\nu_l,o)) \tag{8}$$

$$E(LL_m)=\sum_{i=1}^{N}\sum_{c=1}^{C}\sum_{k=1}^{K}\gamma_{ick}\log(f(m_i\mid c,k,\sigma_m,\nu_m)) \tag{9}$$

Forward-backward algorithm [2] is used to calculate the posterior probability γ_{ick} that the ith exon to be in aberration state c and clonal cluster k.

In the maximization step of the EM algorithm, we define the objective function as $f(\theta)=E(LL_l)+E(LL_m)$ and use Newton–Raphson algorithm to update model parameters until predefined convergence criterion is met. The update process of model parameters from iteration n to n+1 is performed with formula:

$$\theta_{n+1}=\theta_n-\left[Hf(\theta_n)\right]^{-1}\nabla f(\theta_n) \tag{10}$$

For example, during iteration n, the first and second partial derivatives of $f(\theta)$ with respect to parameter o are derived as:

$$\frac{\partial f(\theta)}{\partial o}=\sum_{i=1}^{N}\sum_{c=1}^{C}\sum_{k=1}^{K}\gamma_{ick}\frac{(\nu_l+1)(l_i-\mu_{ck}^l)}{\nu_l\sigma_l^2+(l_i-\mu_{ck}^l)^2} \tag{11}$$

$$\frac{\partial^2 f(\theta)}{\partial^2 o}=\sum_{i=1}^{N}\sum_{c=1}^{C}\sum_{k=1}^{K}\gamma_{ick}\frac{(\nu_l+1)}{(\nu_l\sigma_l+\frac{(l_i-\mu_{ck}^l)^2}{\sigma_l})^2}\left(\frac{2}{\sigma_l^2}(l_i-\mu_{ck}^l)^2-(\nu_l+(\frac{l_i-\mu_{ck}^l}{\sigma_l})^2)\right) \tag{12}$$

We define relative increment of the value of the log-likelihood function of LCR and MAF from iteration n-1 to n as follows:

$$Inc=\frac{2*\left|LL_n-LL_{n-1}\right|}{\left|LL_n\right|+\left|LL_{n-1}\right|} \tag{13}$$

where LL_n is the value of the log-likelihood function in the nth iteration. If the value of Inc is less than a specific threshold ($1\text{x}10^{-4}$), the parameter updating procedure is then stopped.

We use the approach discussed in ref [2] to estimate the initial state distribution π and state transition matrix A. The EM algorithm for CloneCNA is implemented as follows: (1) start with initial parameters $\theta^{(0)}=(\pi^{(0)},A^{(0)},\beta^{(0)},o^{(0)},\sigma_l^{(0)},\nu_l^{(0)},\sigma_m^{(0)},\nu_m^{(0)})$ and calculate the posterior probability $\gamma_{ick}^{(1)}$ using the standard forward-backward algorithm, (2) update $\theta^{(1)}=(\pi^{(1)},A^{(1)},\beta^{(1)},o^{(1)},\sigma_l^{(1)},\nu_l^{(1)},\sigma_m^{(1)},\nu_m^{(1)})$ using Newton–Raphson method, (3) repeat step (1) and (2) until a specified number of iterations are reached or the convergence criterion is met. The converged parameters $\hat{\theta}=(\hat{\pi},\hat{A},\hat{\beta},\hat{o},\hat{\sigma}_l,\hat{\nu}_l,\hat{\sigma}_m,\hat{\nu}_m)$ in the last iteration of the training process will be output as the optimal estimators. The copy numbers and cellularity of each exon are determined by the hidden state associated with the maximum posterior probability. We perform a grid search of θ to find optimal initial parameters.

1.2.3. Bayesian Information Criterion for model selection

To determine the optimal number of clonal clusters, one feasible solution is to perform an exhaustive search for possible values but it is practically less efficient. It is possible to increase the likelihood of the model by adding the number of clonal clusters, but this may result in over-fitting of the model. BIC is effective to solve this kind of problems by introducing a penalty term for the number of free parameters in the model. Thus, we implement a model selection module based on BIC to choose the optimal number of clonal clusters. The BIC of a model is defined as follows:

$$BIC = -\ln \hat{L} + \frac{\alpha}{2} k \ln(n) \tag{14}$$

where $\hat{L}$ is the maximized likelihood value of the model, $\alpha > 0$ is the regularizing term, k is the number of free parameters to be estimated and n is the number of exons. We aim to find an optimal value of K that leads to the model associated with the lowest value of BIC. We present the model selection procedure as follows: CloneCNA starts with the initial assumption of tumor homogeneity ($K = 1$) and then iteratively increases clonal cluster count by one until the BIC value of the model no longer decreases.

We do not directly calculate the BIC of the model, but the difference of BIC between two adjacent models. Suppose that the number of clonal clusters is i in the ith iteration, and the BIC difference between the current model and the model in the last iteration is defined as follows:

$$dBIC = BIC_i - BIC_{i-1} = -(\ln \hat{L}_i - \ln \hat{L}_{i-1}) + \frac{\alpha}{2}(k_i - k_{i-1})\ln(n) = -\Delta\hat{L}_i + \frac{\alpha}{2}\Delta k_i \ln(n) \tag{15}$$

where $\Delta\hat{L}_i$ is the increment of the likelihood function from iteration i-1 to i, and Δk_i is the increased number of free parameters and calculated as:

$$\Delta k_i = (2i-1)(C-1)^2 + 2(C-1) + 1 \tag{16}$$

where C is the number of copy number aberration states defined in Table S1. The iteration is stopped once the BIC difference is greater than zero.

1.3. Reliability score for CNA detection

To evaluate the reliability of CloneCNA results, we provide a measurement to depict how well the data fit to the model. The probability densities of the regions of which the LCR and MAF signals do not fit to the HMM used in CloneCNA should be much lower than those of other regions, which inspires us to use the probability densities of observed LCR and MAF signals to measure reliability. To make the reliability scores comparable among different hidden states in the HMM, we further divide the probability densities of the LCR and MAF signals by the probability densities of expected LCR and MAF signals. Accordingly, we define a reliability score for each segmented region in the results as follows:

$$Score_i = mean\left(\frac{p(l_{ij} \mid c,k,\sigma_l,\nu_l,o)}{p(\bar{l} \mid c,k,\sigma_l,\nu_l,o)} \frac{p(m_{ij} \mid c,k,\sigma_m,\nu_m)}{p(\bar{m} \mid c,k,\sigma_m,\nu_m)} \right) \tag{17}$$

where l_{ij} and m_{ij} are the LCR and MAF values of the jth exon in the ith region, and $\bar{l}$ and $\bar{m}$ are the expected LCR and MAF values associated with aberration state c and clonal cluster k.

1.4. Performance evaluation

To evaluate CNA detection performance, the CNA calls of all exons are used as the golden standard to compare the abilities of different computational methods. Accordingly, for simulated tumor samples, the CNA calls of all exons pre-determined in simulation experiment are treated as the ground truth. For the TNBC samples also assayed by Affymetrix SNP6.0 array, we fetch the exons covered by ASCAT (version 2.1) [3] analysis of tumor SNP array data, and use the CNA calls of these exons as the ground truth. Using array-based results as the gold standard has been adopted in similar studies [4-6] in order to measure prediction performance with next-generation sequencing data. ASCAT is one of the state-of-the-art method designed for array-based platforms [6, 7], therefore we followed the same approaches and used ASCAT results on TNBC samples as the ground truth. We perform performance evaluation by comparing the results of the computational methods investigated in this study to the ground truth in terms of sensitivity, specificity and accuracy.

Exons with copy number alteration (copy number $\neq$ 2) are treated as positives, and copy neutral (copy number = 2) exons are treated as negatives. For each tumor sample, true positives (TP) are defined as positive exons that are correctly detected as positives by a computational method, true negatives (TN) are defined as negative exons that are correctly detected as negatives, false positives (FP) are defined as negative exons that are wrongly detected as positives, and false negatives (FN) are defined as positive exons that are wrongly detected as negatives. Three performance measurements, sensitivity, specificity and accuracy, are employed to compare the performance of CNA detection for different methods, which are defined as follows:

$$sensitivity = \frac{TP}{TP + FN} \quad (18)$$

$$specificity = \frac{TN}{TN + FP} \quad (19)$$

$$accuracy = \frac{TP + TN}{TP + FP + TN + FN} \quad (20)$$

Since THetA and CloneCNA model multiple clones when analyzing WES data of tumors, different copy numbers may be represented in different clones for an exon, therefore we consider an exon to be negative if all tumor cells are predicted to have a neutral copy in the exon, otherwise it is treated as positive.

1.5. Simulation of intra-tumor heterogeneity

To simulate tumors containing multiple subclones, we first manually generate four tumor genomes (denoted as *t1*, *t2*, *t3* and *t4*), and mix different combinations of genomes at predefined proportions. As illustrated in Figure S1, the tumor genomes are generated by dividing the reference genome into a number of segments, and each segment is assigned with a specific genomic aberration defined by total copy number and major allele copy number. Each genome is constructed by introducing new genomic aberrations based on the previously generated genome. For example, genome *t2* is built from genome *t1*, genome *t3* is built from genome *t2*, and so on. The WES data of a real normal sample is used to generate sequencing data of the test genomes by following these steps: 1) For each segment of the test genome, reads aligned to the region are randomly and repeatedly sampled from BAF file of the normal sample according to the copy number of the segment, 2) nucleotide sequences of sampled reads are properly modified to match the BAF of SNPs within the segment, and 3) reads are merged to generate BAM by using SAMtools. To simulate normal cell contamination, we also generate a normal genome (denoted as *n*) by following the same procedure. The aberration information of all genomes is provided in Table S1. We generate mixtures of genomes by sampling reads from BAM files at predefined proportions, and reads are sampled to 100X coverage at all mixtures.

For each simulated sample, the underlying cellularity of clonal clusters are computed by using contribution from each tumor genome making up the mixture. For example, for the mixture "t1_015t2_010t3_015n_060" made up by genomes *t1*, *t2*, *t3* and *n* with corresponding proportion of p1=0.15, p2=0.10, p3=0.15 and p4=0.6 respectively, then the underlying cellularity of all clonal cluster equal to 0.15 (p3), 0.25 (p3+p2) and 0.4 (p3+p2+p1).

1.6. Details of the investigated methods

When running all the investigated methods on the simulated samples, the real normal sample used to generate simulated tumor samples is treated as the paired normal sample for all simulated tumor samples. The other details of all methods are provided in the following sections.

1.6.1. ExomeCNV

ExomeCNV-1.4 [8] takes depth of coverage files of paired tumor-normal samples as inputs and outputs detected CNAs. For each pair of tumor-normal samples, the coverage files are generated using GATK's DepthOfCoverage [9]. We run ExomeCNV with parameters set to their default values as described in the ExomeCNV documentation.

1.6.2. Control-FREEC

Control-FREEC-8.1 [4] first infers the copy numbers of genomic segments using paired tumor-normal samples, then employs Gaussian mixture model (GMM) to identify tumor genotypes with correction for both normal cell contamination and tumor aneuploidy. We use the option "ploidy=2, contaminationAdjustment=TRUE, readCountThreshold=10" for simulated samples, and use the option "ploidy=2,3,4, contaminationAdjustment=TRUE, readCountThreshold=10" for real samples.

1.6.3. EXCAVATOR

We use EXCAVATOR-2.2 [10] to detect CNAs with the option "--mode somatic". For both simulated and real data, we use the "TargetPerla.pl" module to calculate GC content and mappability values of target regions with the option "--assembly hg18".

1.6.4. THetA

We use THetA-0.62 [11] to estimate tumor purities and detect CNAs with the option "--NUM_PROCESSES 20, --MAX_NORMAL 0.9, --MAX_K 6" for simulated data. To avoid prohibitively long running time, we use the option "--NUM_PROCESSES 20, --MAX_NORMAL 0.5, --MAX_K 3" for real TNBC

samples. In addition, we are able to run THetA for up to two tumor populations due to time complexity limitations, which is also the default setting as described in THetA documentation. Since THetA generally outputs multiple solutions, we select the default best solution whenever this happens, as recommended by THetA. Moreover, the segmentation files for THetA are obtained by using EXCAVATOR software.

1.6.5. CloneCNA

CloneCNA takes about 10 minutes to run a tumor WES data, using a standard desktop PC with 3.4GHz CPU and 4G RAM. ExomeSeqMiner software is used to prepare input files for CloneCNA and developed in-house, it currently works on Unix-based systems. It is required that MATLAB or MATLAB Compiler Runtime (MCR) installed on users' computers to run CloneCNA software. For both simulated and real data, we fetch the read counts of exons with the option "--mapQ 20", and obtain the read depths of SNPs with the option "--baseQ 10, --minDepth 10".

Reference

1. Li H, Handsaker B, Wysoker A, Fennell T, Ruan J, Homer N, Marth G, Abecasis G, Durbin R: **The sequence alignment/map format and SAMtools**. *Bioinformatics* 2009, **25**(16):2078-2079.
2. Rabiner LR: **A tutorial on hidden Markov models and selected applications in speech recognition**. *Proceedings of the IEEE* 1989, **77**(2):257-286.
3. Van Loo P, Nordgard SH, Lingjærde OC, Russnes HG, Rye IH, Sun W, Weigman VJ, Marynen P, Zetterberg A, Naume B: **Allele-specific copy number analysis of tumors**. *Proceedings of the National Academy of Sciences* 2010, **107**(39):16910-16915.
4. Boeva V, Popova T, Bleakley K, Chiche P, Cappo J, Schleiermacher G, Janoueix-Lerosey I, Delattre O, Barillot E: **Control-FREEC: a tool for assessing copy number and allelic content using next-generation sequencing data**. *Bioinformatics* 2012, **28**(3):423-425.
5. Ha G, Roth A, Lai D, Bashashati A, Ding J, Goya R, Giuliany R, Rosner J, Oloumi A, Shumansky K: **Integrative analysis of genome-wide loss of heterozygosity and monoallelic expression at nucleotide resolution reveals disrupted pathways in triple-negative breast cancer**. *Genome research* 2012, **22**(10):1995-2007.
6. Favero F, Joshi T, Marquard AM, Birkbak NJ, Krzystanek M, Li Q, Szallasi Z, Eklund AC: **Sequenza: allele-specific copy number and mutation profiles from tumor sequencing data**. *Annals of Oncology* 2015, **26**(1):64-70.
7. Liu Y, Li A, Feng H, Wang M: **TAFFYS: An Integrated Tool for Comprehensive Analysis of Genomic Aberrations in Tumor Samples**. *PloS one* 2015, **10**(6):e0129835.
8. Sathirapongsasuti JF, Lee H, Horst BA, Brunner G, Cochran AJ, Binder S, Quackenbush J, Nelson SF: **Exome sequencing-based copy-number variation and loss of heterozygosity detection: ExomeCNV**. *Bioinformatics* 2011, **27**(19):2648-2654.
9. McKenna A, Hanna M, Banks E, Sivachenko A, Cibulskis K, Kernytsky A, Garimella K, Altshuler D, Gabriel S, Daly M: **The Genome Analysis Toolkit: a MapReduce framework for analyzing next-generation DNA sequencing data**. *Genome research* 2010, **20**(9):1297-1303.
10. Magi A, Tattini L, Cifola I, D'Aurizio R, Benelli M, Mangano E, Battaglia C, Bonora E, Kurg A, Seri M: **EXCAVATOR: detecting copy number variants from whole-exome sequencing data**. *Genome biology* 2013, **14**(10):R120.
11. Oesper L, Mahmoody A, Raphael BJ: **THetA: inferring intra-tumor heterogeneity from high-throughput DNA sequencing data**. *Genome Biol* 2013, **14**(7):R80.
