# Supplementary material for: CloneCNA: detecting subclonal somatic copy number alterations in heterogeneous tumor samples from whole-exome sequencing data
Source: BMC Bioinformatics. 2016 Aug 19;17:310. doi: 10.1186/s12859-016-1174-7 (PMC4990858; doi:10.1186/s12859-016-1174-7)
Supplement: Additional file 2: — Presentations of Supplementary Figures. (PDF 1712 kb) [file 12859_2016_1174_MOESM2_ESM.pdf]

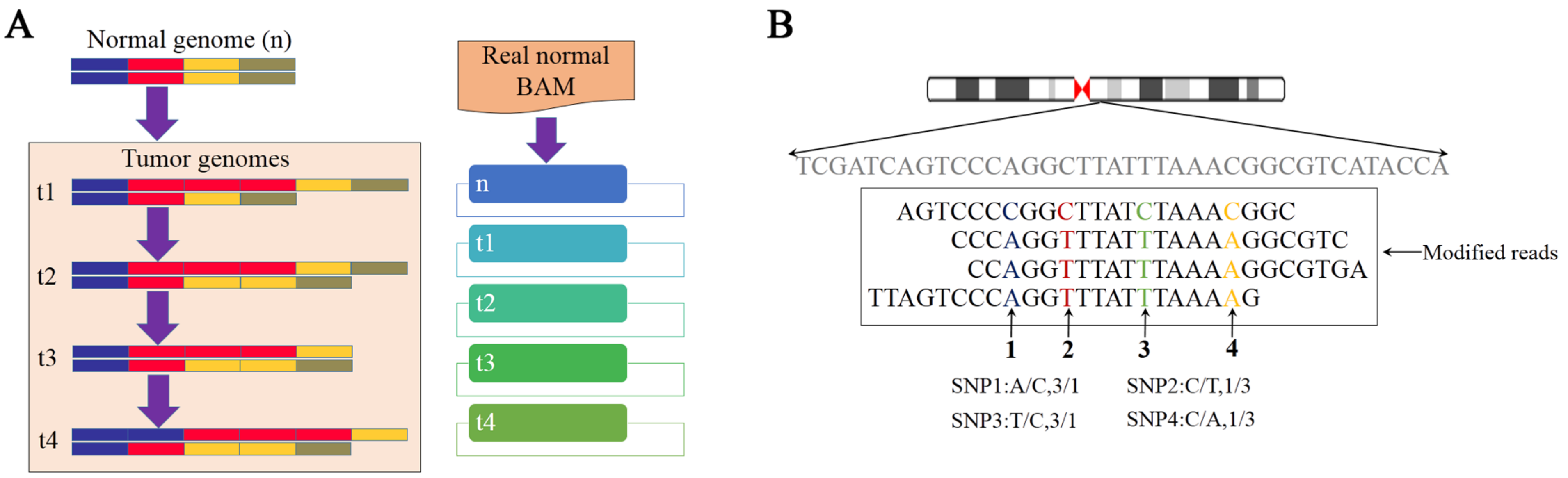

Figure S1. Simulation of intra-tumor heterogeneity. **A.** Process of constructing test genomes. Four tumor genomes and one normal genome are generated. Each genome contains a number of segments, each of which is assigned with a specific genomic aberration defined by total copy number and major allele copy number. Each genome is constructed by introducing new genomic aberrations based on the previously generated genome. **B.** Process of generating sequencing data. Reads are randomly sampled from BAF file of a normal sample and further processed to match the BAF of SNPs within each segment. SNP is displayed as: reference allele/alternative allele, reference allele copy number/alternative allele copy number.

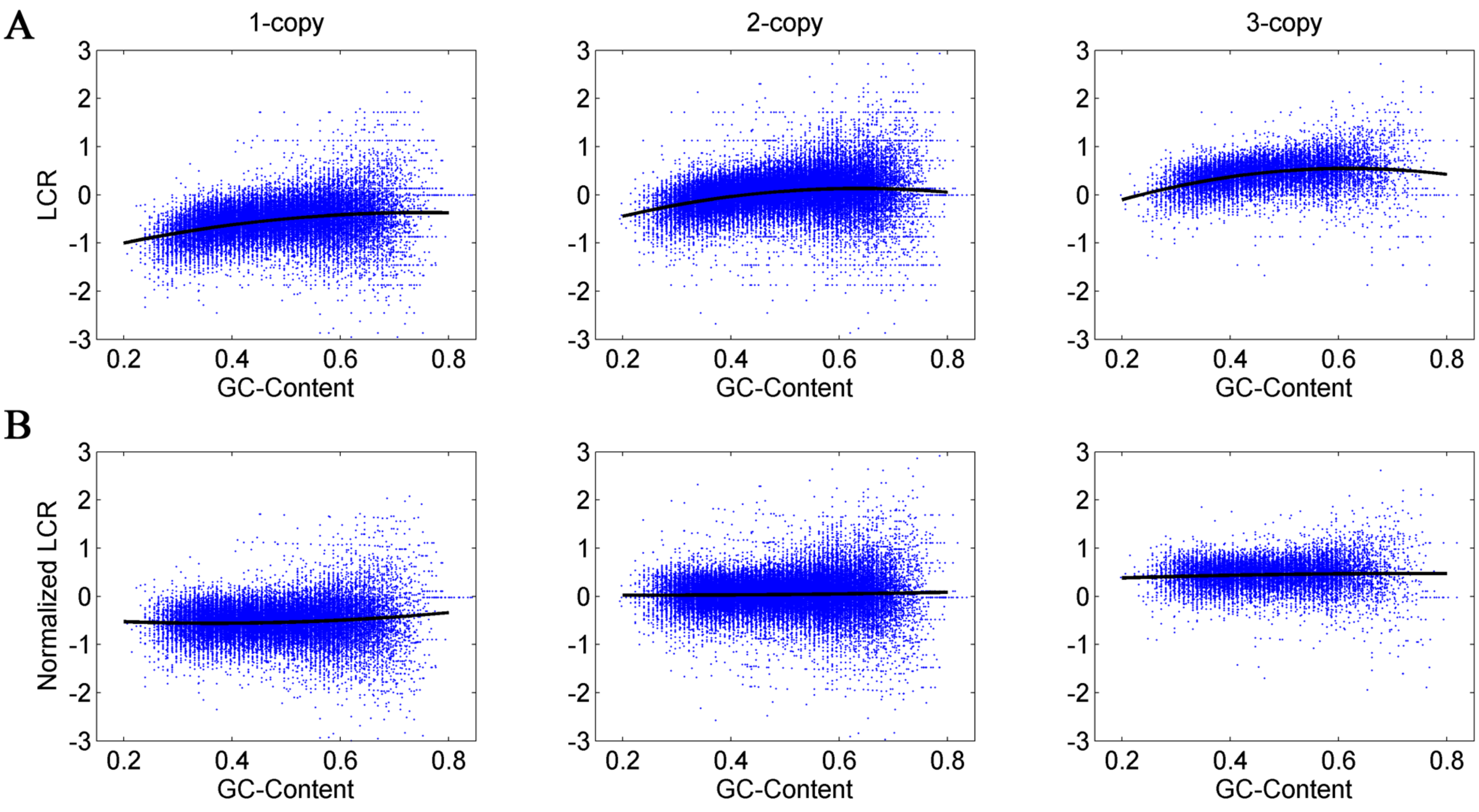

Figure S2. Plots of original and normalized LCR signals with respect to GC-Content. A. Plots of original LCR signals. B. Plots of corrected LCR signals. Relationship between LCR and GC-Content is fitted by a cubic polynomial curve for 1, 2, 3-copy region. It is observed that GC-content bias is significantly eliminated after normalization.

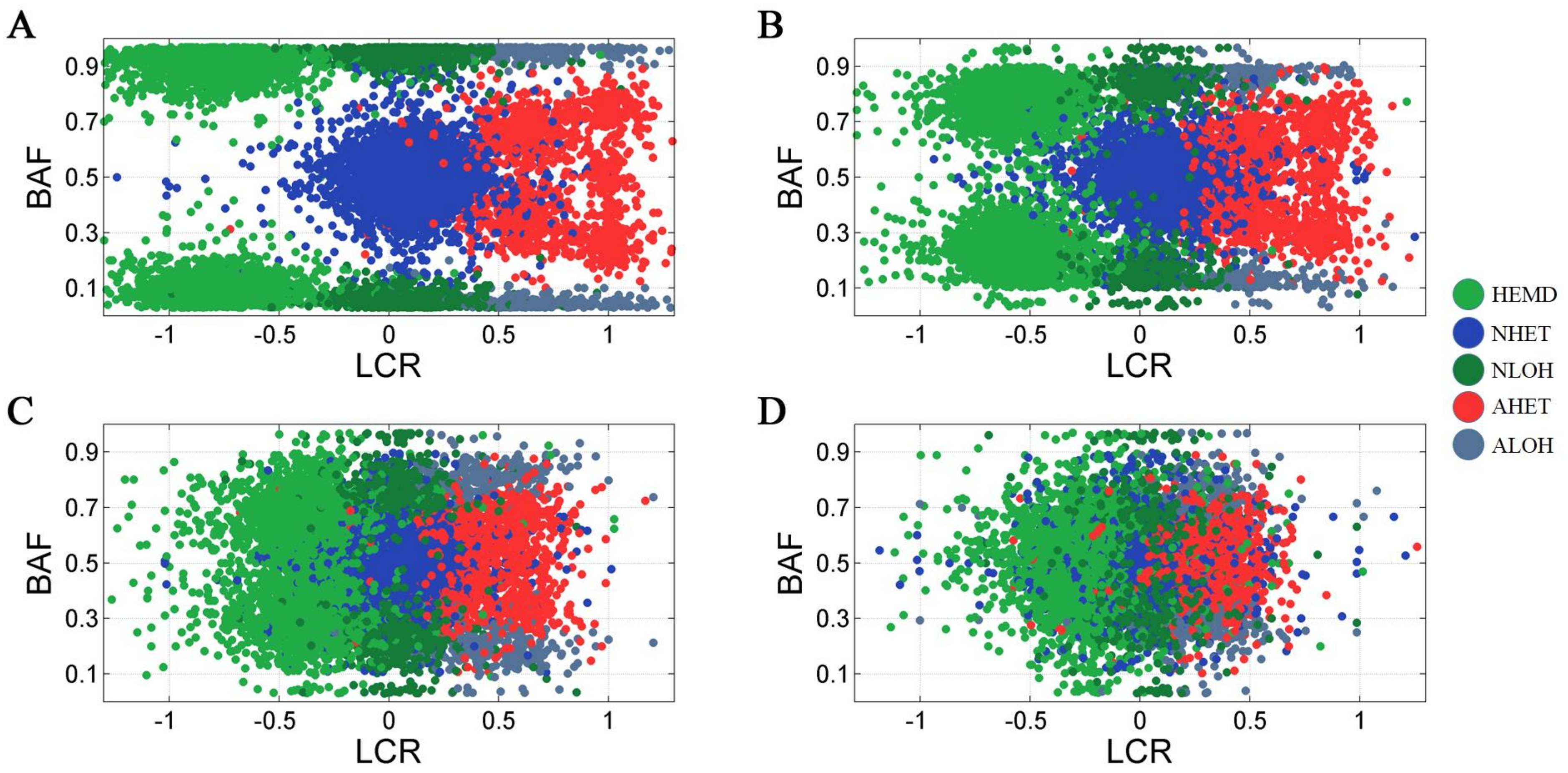

Figure S3. The influence of normal cell contamination for LCR and BAF signals associated with different types of aberrations. Results on sample with tumor purity of 0.9 (**A**), 0.7 (**B**), 0.5 (**C**) and 0.3 (**D**) are shown respectively. For high tumor purity of 0.9, distinct clusters of LCR and BAF signals are including hemizygous deletion (HEMD), copy neutral heterozygous (NHET), copy neutral LOH (NLOH), amplified heterozygous (AHET) and amplified LOH (ALOH) are separated with visible boundaries. When tumor purity decreases, it is observed that for each aberration both the LCR and BAF signals gradually approach close to the expected values of normal genotype (0.5 for BAF and 0 for LCR).

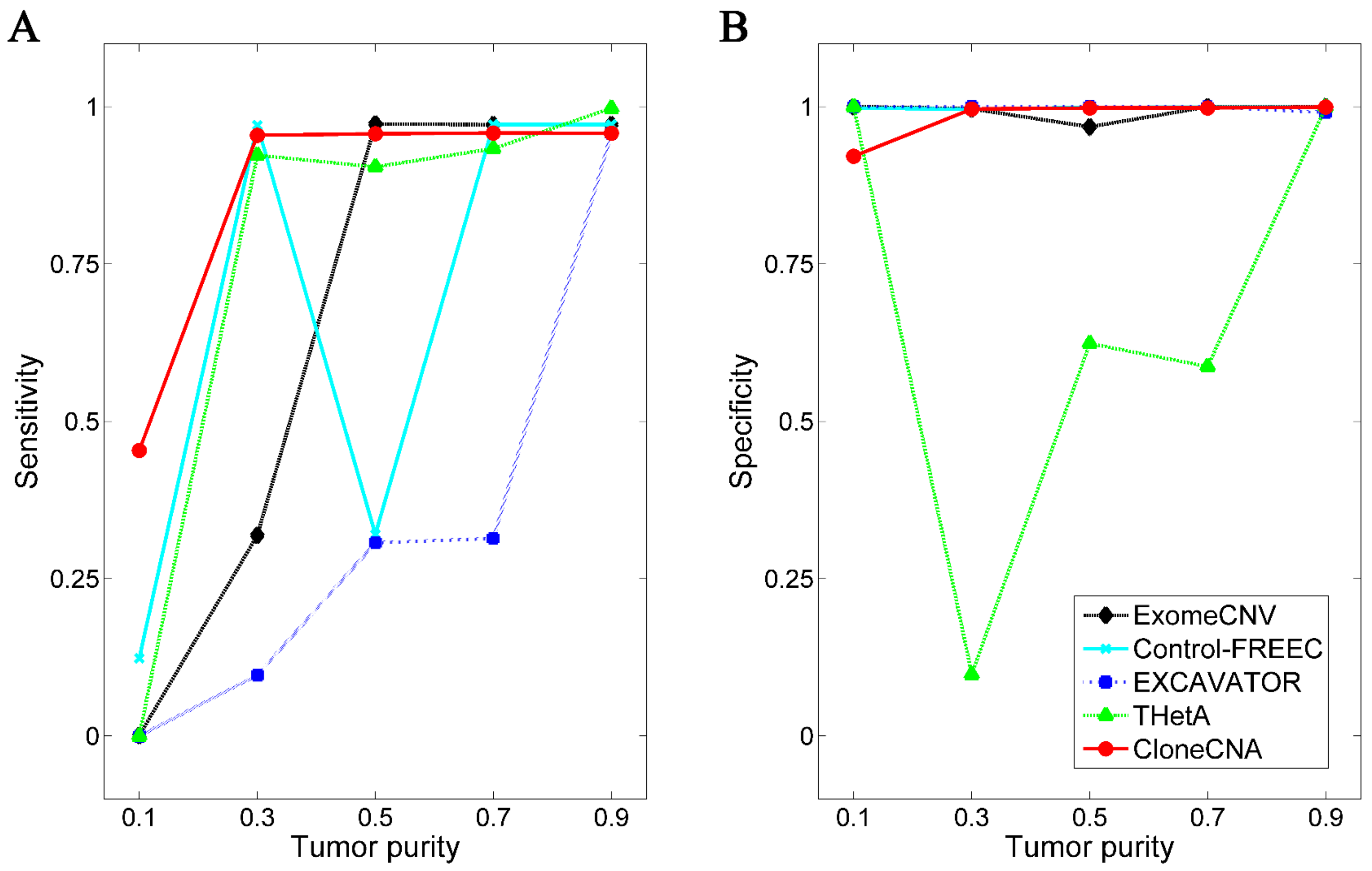

Figure S4. CNA detection performance of ExomeCNV, Control-FREEC, EXCAVATOR, THetA and CloneCNA on simulated homogeneous tumor samples.

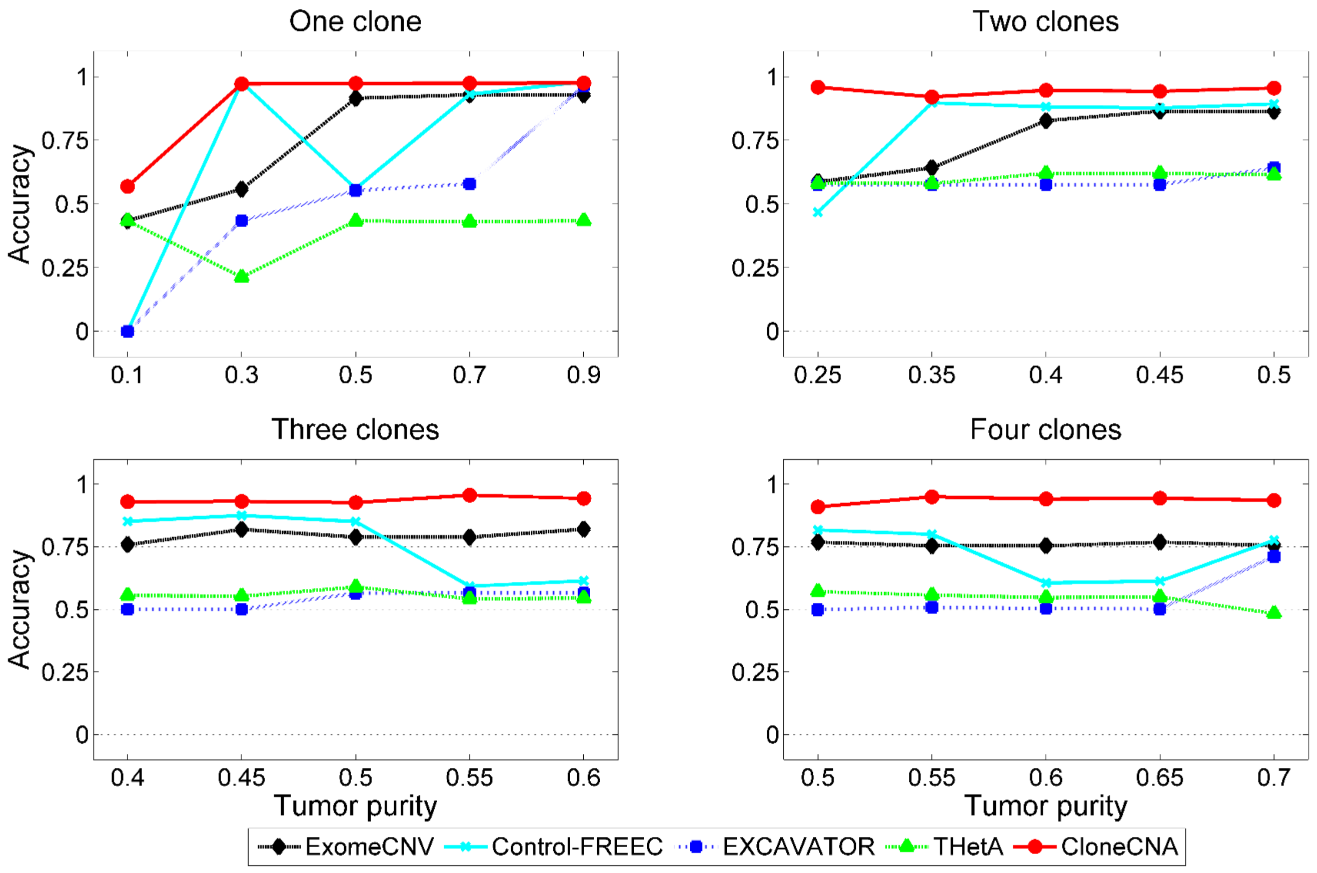

Figure S5. Copy number prediction performance on simulated samples. Accuracy is measured based on the prediction results of each investigated method.

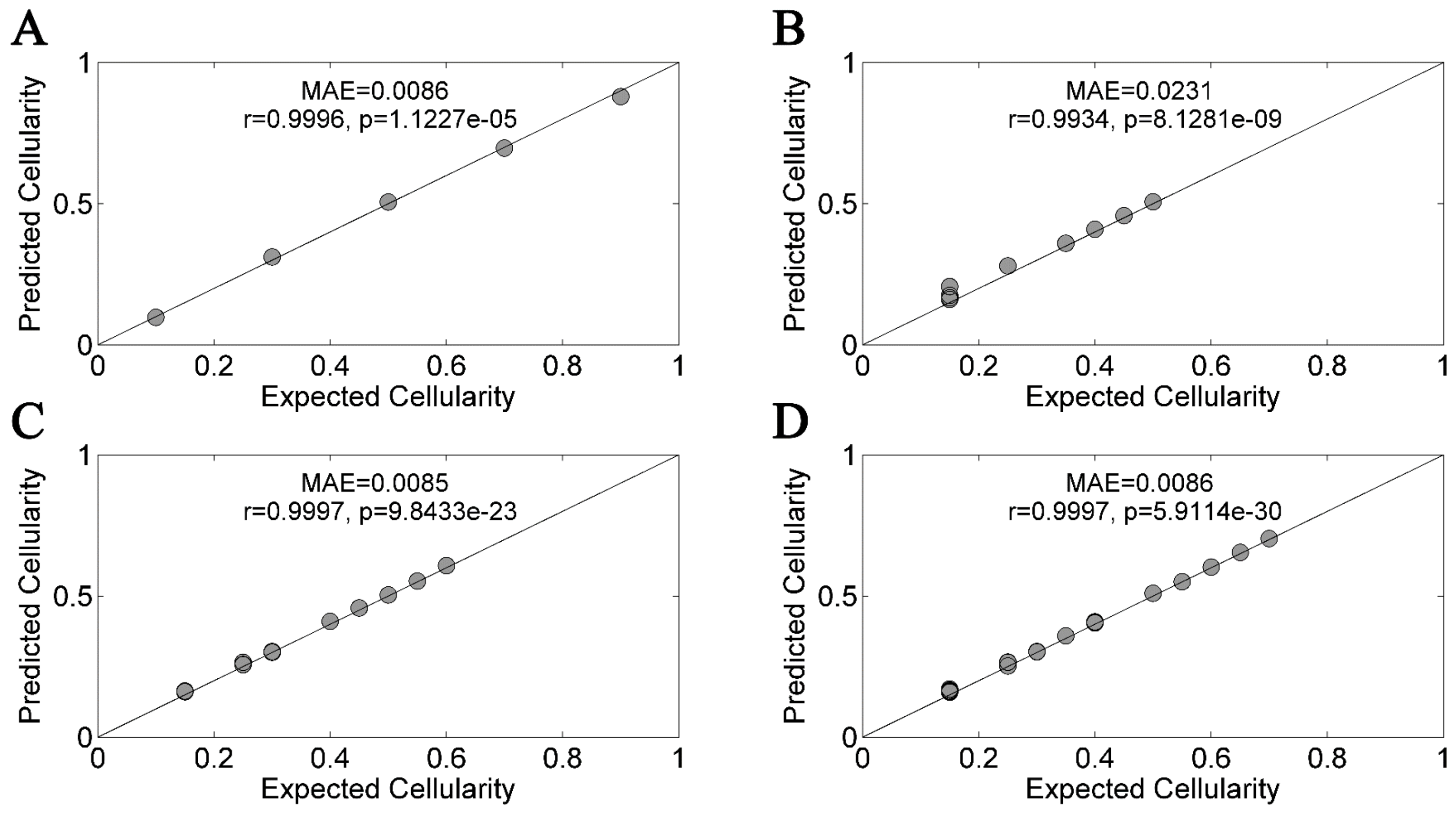

Figure S6. Cellularity prediction performance of CloneCNA on simulated samples. Estimated cellularity is compared to the underlying ground truth cellularity for each simulated sample. Results on samples containing one (**A**), two (**B**), three (**C**) and four (**D**) tumor clones are shown respectively.

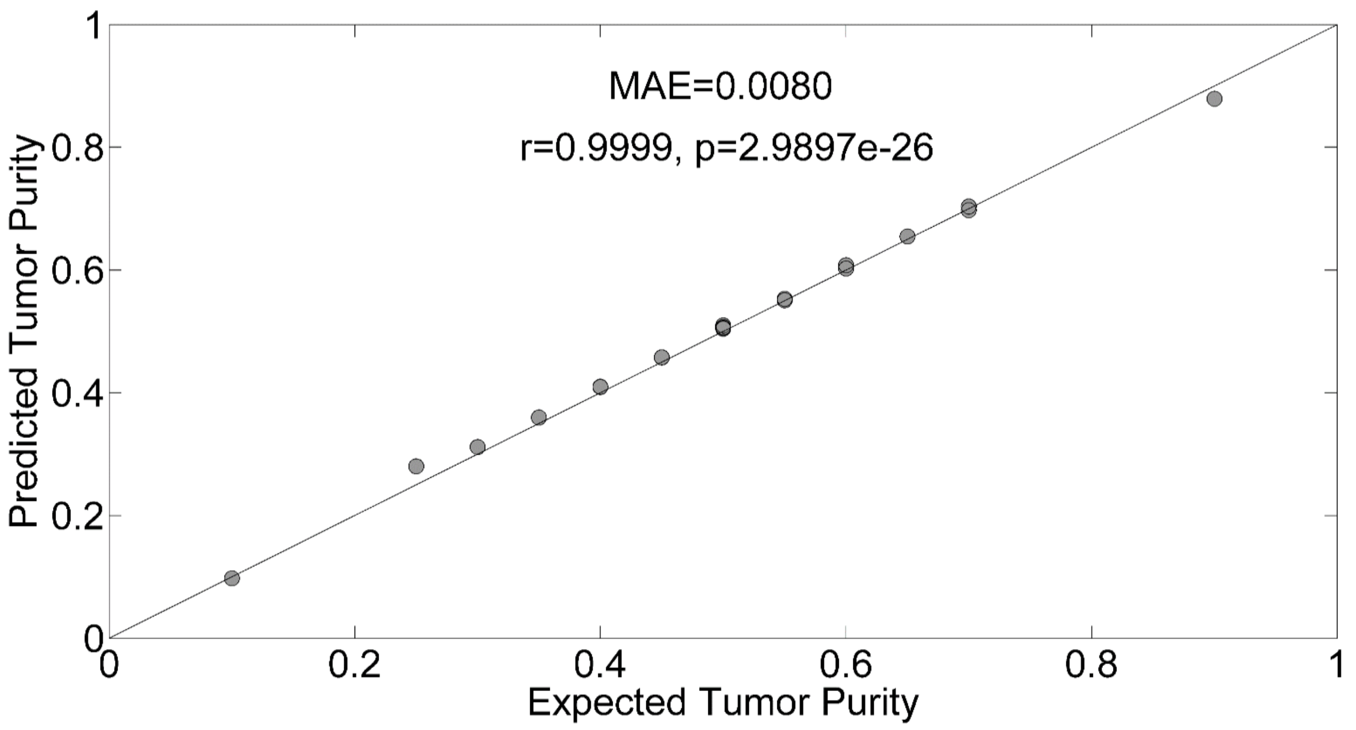

Figure S7. Tumor purity estimation results of CloneCNA on simulated samples. Estimated tumor purity on each simulated sample is compared to the expected tumor purity.

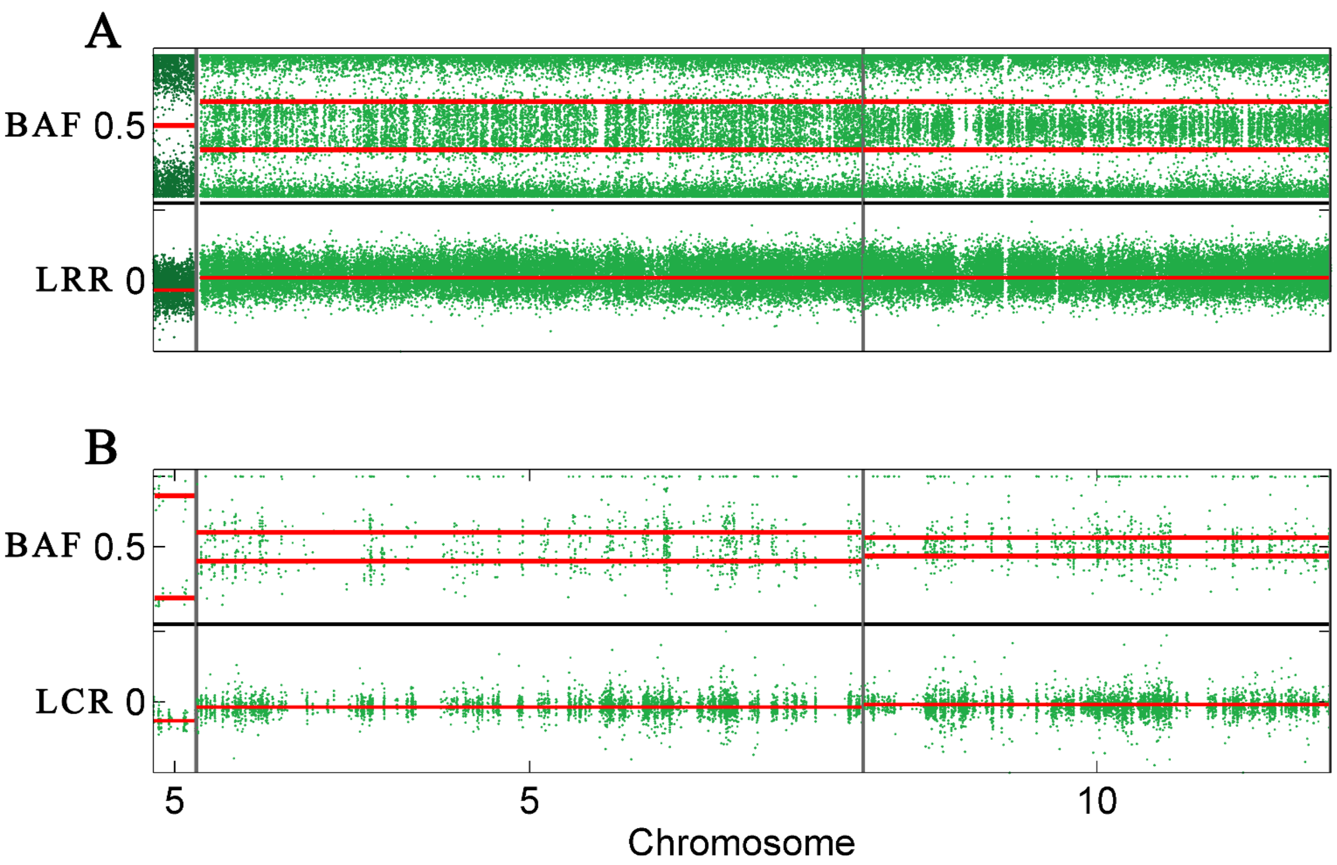

Figure S8. Illustration of intra-tumor heterogeneity of sample SA052. **A.** The homozygous and hemizygous deletions predicted by ASCAT on chromosome 5p, 5q and 10q. Distinct mismatching between the mean of observed BAF signals and the expected BAF value is observed. **B.** CloneCNA results on chromosome 5p, 5q and 10q. The hemizygous deletions predicted by ASCAT are represented in subclonal clusters from the results of CloneCNA. In addition, CloneCNA identifies a clonal hemizygous deletion on chromosome 5p.

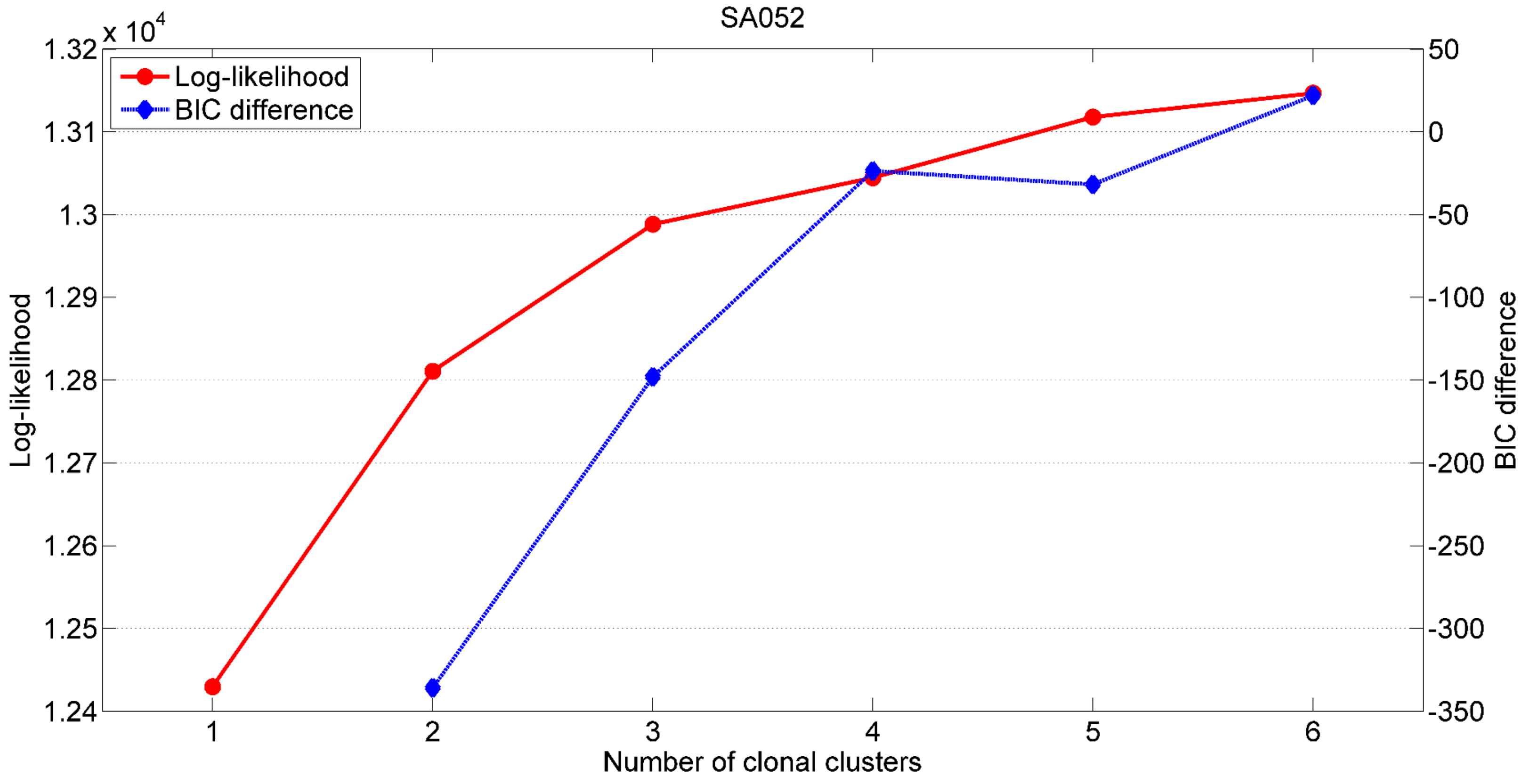

Figure S9. The log-likelihoods and BIC differences of sample SA052 under different number of clonal clusters. CloneCNA infers K = 5 as the optimal number of clonal clusters.

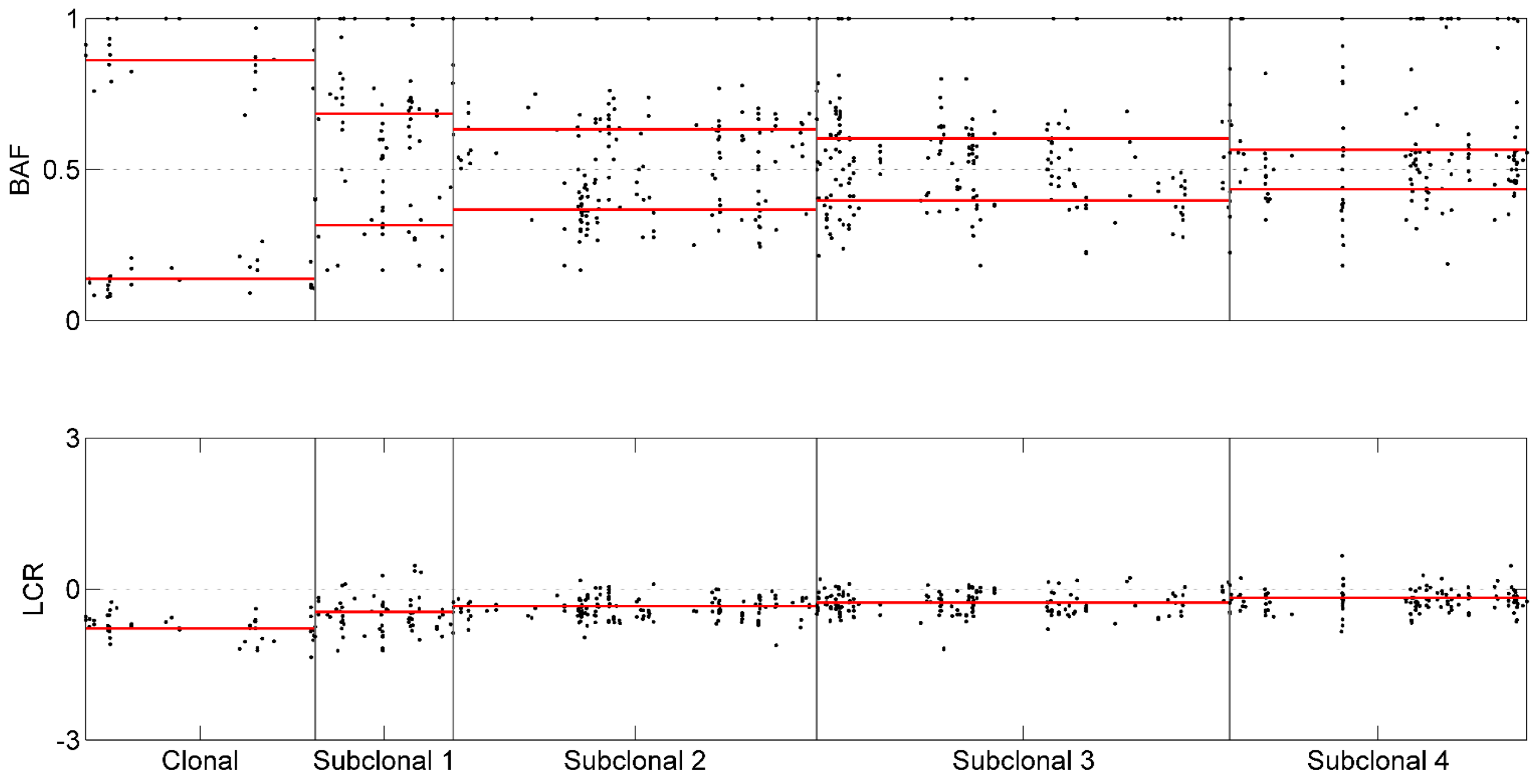

Figure S10. BAF and LCR signal distributions in hemizygous deletion regions represented in clonal and subclonal clusters.

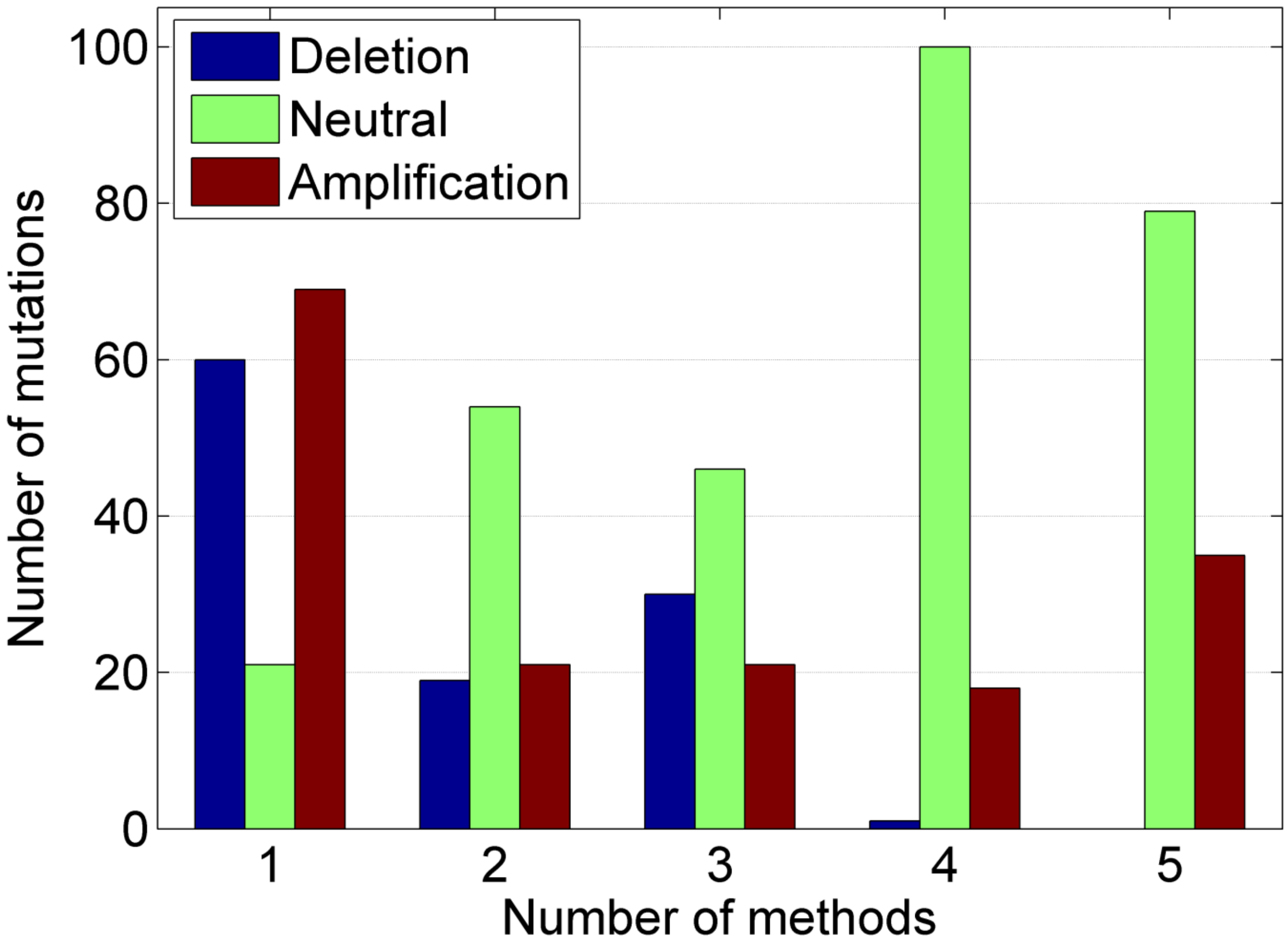

Figure S11. The number of mutations commonly predicted to be in copy deleted, neutral and amplified regions by multiple methods.
